# A multiparameter liquid biopsy approach allows to track melanoma dynamics and identify early treatment resistance

**DOI:** 10.1038/s41698-024-00567-0

**Published:** 2024-03-28

**Authors:** Maria Chiara Scaini, Cristina Catoni, Cristina Poggiana, Jacopo Pigozzo, Luisa Piccin, Kevin Leone, Ilaria Scarabello, Antonella Facchinetti, Chiara Menin, Lisa Elefanti, Stefania Pellegrini, Valentina Aleotti, Riccardo Vidotto, Francesca Schiavi, Alessio Fabozzi, Vanna Chiarion-Sileni, Antonio Rosato

**Affiliations:** 1 Immunology and Molecular Oncology Unit, Veneto Institute of Oncology - IOV IRCCS, Padua, Italy; 2 Medical Oncology 2, Veneto Institute of Oncology - IOV IRCCS, Padua, Italy; 3 Department of Surgery, Oncology and Gastroenterology (DiSCOG), Oncology Section, University of Padua, Padua, Italy; 4 Familial Cancer Clinic, Veneto Institute of Oncology - IOV IRCCS, Padua, Italy; 5 Oncology Unit 3, Veneto Institute of Oncology - IOV IRCCS, Padua, Italy

**Keywords:** Prognostic markers, Molecular medicine

## Abstract

Melanoma heterogeneity is a hurdle in metastatic disease management. Although the advent of targeted therapy has significantly improved patient outcomes, the occurrence of resistance makes monitoring of the tumor genetic landscape mandatory. Liquid biopsy could represent an important biomarker for the real-time tracing of disease evolution. Thus, we aimed to correlate liquid biopsy dynamics with treatment response and progression by devising a multiplatform approach applied to longitudinal melanoma patient monitoring. We conceived an approach that exploits Next Generation Sequencing (NGS) and droplet digital PCR, as well as the FDA-cleared platform CellSearch, to analyze circulating tumor DNA (ctDNA) trend and circulating melanoma cell (CMC) count, together with their customized genetic and copy number variation analysis. The approach was applied to 17 stage IV melanoma patients treated with BRAF/MEK inhibitors, followed for up to 28 months. BRAF mutations were detected in the plasma of 82% of patients. Single nucleotide variants known or suspected to confer resistance were identified in 70% of patients. Moreover, the amount of ctDNA, both at baseline and during response, correlated with the type and duration of the response itself, and the CMC count was confirmed to be a prognostic biomarker. This work provides proof of principle of the power of this approach and paves the way for a validation study aimed at evaluating early ctDNA-guided treatment decisions in stage IV melanoma. The NGS-based molecular profile complemented the analysis of ctDNA trend and, together with CMC analysis, revealed to be useful in capturing tumor evolution.

## Introduction

Melanoma accounts for over 80% of skin cancer-related deaths, despite representing only 1% of all skin tumors. However, the mortality rate has fallen since 2011 owing to the approval of numerous new targeted or immunotherapy agents^1^. Indeed, the systemic treatment of metastatic melanoma has radically changed due to an improvement in the understanding of its genetic landscape. In this regard, the identification of molecular predictive factors has become of paramount importance for treatment choices in patients with stage III or IV disease^2^. Combined blockade in patients harboring a BRAF p.V600E/K mutation has led to significant improvements in overall survival (OS) in adjuvant and advanced settings^2,3^. Nevertheless, the complete response to BRAF/MEK inhibitor targeted therapy (BRAFi/MEKi) is only transitory in approximately 50% of cases. Therefore, many efforts have been made in recent years to identify the mechanisms underlying the switch from response to resistance^4^. The main problem lies in the great heterogeneity of the disease at inter- and intra- metastatic level^5–8^, which hinders the identification of reliable biomarkers to monitor therapy response^5^. The recent development of highly sensitive techniques applied to circulating tumor cells (CTCs) and cell-free circulating DNA (cfDNA) has overcome the problems associated with the analysis of a single biopsy, resulting in unprecedented resolution of tumor heterogeneity^9–12^. Circulating melanoma cell (CMC) detection could be indicative of subclinical disease and metastatic spread, while their enumeration and genetic analysis (although challenging) could be useful to assess the response/sensitivity to specific drugs^5,13–17^. Although limited, studies with the CMC kit for the FDA-cleared CellSearch platform showed similar results, that is the identification of two or more CMCs in 7.5 ml of blood in up to 40% of patients with advanced melanoma, and an association of CMC detection with overall survival (OS)^15,18–20^. Furthermore, several studies have shown that circulating tumor DNA (ctDNA) is a good biomarker for follow-up and early detection of progression in patients with metastatic cancer^21–24^. As ctDNA derives from tumor cells, it can reflect the mutational burden and could identify potential druggable targets, even when the tumor is not per se accessible^20^. In the melanoma setting, ctDNA is commonly used as a tracer of single driver mutations that have been previously identified in the cancer tissue of the same patient. This type of analysis, which is increasingly used in routine screening, aims to monitor cancer progression, response to therapy, and resistance onset^25–30^. Nevertheless, the extreme hypermutability and heterogeneity of the disease pushes in the direction of a more complete and exhaustive description of the genomic landscape, which can possibly be able to reliably define the dynamics of the systemic disease^31^. Next Generation Sequencing (NGS) can provide a comprehensive ctDNA mutation profile, and in turn, the detection of mutations potentially not present in the primary tumor could help in defining tumor clonal evolution in individual patients. Although this approach is technically challenging owing to the limited quantity and poor quality of the highly fragmented cfDNA^32,33^, there is a plethora of commercially available panels that are optimized for cfDNA, which typically cover common mutations in driver genes together with hotspots in tumor suppressor- and onco-genes^32,34^. This type of panels tends to exclude genes exclusively associated with melanoma resistance to treatment and/or genes only rarely involved in tumor progression; moreover, panels designed to investigate only specific hotspots do not allow the discovery of novel resistance mutations^35^. Thus far, only a few studies have exploited the potential of targeted melanoma panels optimized for liquid biopsy, i.e. custom melanoma NGS panels^33,36^ or systems based on mutation detection by mass spectrometry^5,37,38^.

**Fig. 1:**
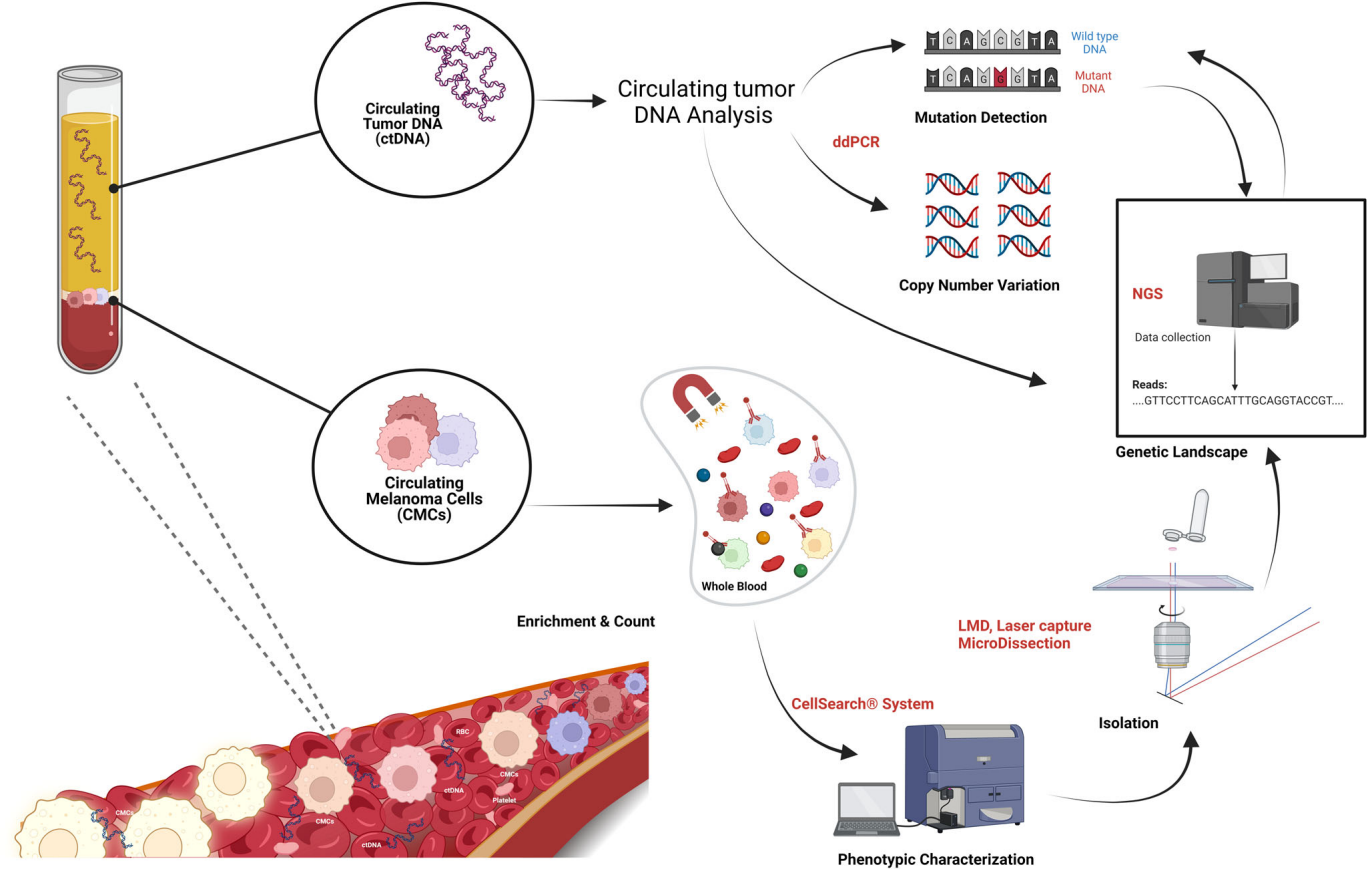
Multiplatform approach overview. To longitudinally track the evolution of the disease, a multiplatform approach was devised that encompasses the use of ddPCR, NGS and CellSearch system on serial blood samples. Both cfDNA and CMCs were analyzed at different time points from the same blood specimen. BRAF/pTERT mutant allele fractions were longitudinally tracked by ddPCR as biomarkers of disease evolution and response to treatment, while a customized NGS panel was exploited to identify tumor genetic landscape and mutations responsible for resistance. In parallel, CMCs were phenotypically characterized and counted through the CellSearch System, and then isolated by laser capture microdissection for genetic landscape identification through NGS analysis. The workflow was created with BioRender (https://biorender.com/).

Here, we present a longitudinal screening strategy applied to a small cohort of stage IV melanoma patients under BRAFi/MEKi, which turned out to be highly successful in monitoring tumor evolution through liquid biopsy analysis. In particular, we developed a multiplatform approach based on the use of CellSearch, NGS, and droplet digital PCR (ddPCR), which allows CMC count, ctDNA tracking, and customized genetic analysis (optimized for low-quality DNA) at every time point (Fig. 1). This study provides proof of principle of the strength of this analysis, its translational validity, and clinical impact, and reveals that it can supply complementary real-time information useful for assessing disease evolution and response. Finally, this kind of combined strategy and, more importantly, the timing of application opens up new scenarios for the management and real-time monitoring of melanoma patients.

## Results

### Study patients and samples

The 17-patient cohort (median age 64) consisted of 29.4% of patients younger than 50, 29.4% between 50 and 64, 17.6% between 65 and 75, and 23.5% over 75 years. Fifty plasma samples were collected during the longitudinal monitoring. Of these, 35 came from patients carrying the BRAF p.V600E mutation, 12 from those with the p.V600K mutation, and 3 from a patient with the rare p.T599dup mutation (Table 1). All patients were enrolled at the beginning of the dabrafenib/trametinib targeted therapy and were followed longitudinally at T1 (month 6), T2 (month 10), and progression (P), whenever this last point did not match with one of the previous points (median follow-up, 480 days; range: 77–853). ctDNA tracking, CMC count, and analysis of the tumor genetic landscape were performed at each time point. More in detail, ctDNA and CMC analyses were carried out at baseline in 100% (17/17) and 94% (16/17) of patients, respectively, whereas at progression the proportion was 78.6% (11/14) for both of them (Table 2, Supplementary Table 1 and Supplementary Fig. 1). The median time of the first evaluation point (T1) and progression (P) was 175 and 364 days, respectively.

**Table 1:** Characteristics of patients enrolled in the study

Patient ID	Sex	Age	Mutation^a^	Disease sites	Stage IV subcategory	Best response (RECIST)	EP	Progression sites	Survival status	Number of samples
1	F	75	p.V600E	Brain, lymph nodes, lung	M1d	PR	N	Brain	Dead	3
3	F	81	p.V600K	Liver	M1c	SD	N	Liver	Dead	3
6	F	42	p.V600E	Peritoneum	M1c	PR	N	Peritoneum	Dead	4
8	M	85	p.V600E	Brain, liver, lymph nodes, lung	M1d	PR	N	Brain, lung, soft tissues, lymph nodes	Dead	3
9	F	37	p.V600E	Soft tissue, lymph nodes, liver, adrenal gland, bones, peritoneum	M1c	PR	N	Soft tissues, liver, heart	Dead	4
10	F	65	p.V600K^b^	Brain, lymph nodes, lung, liver	M1d	PR	N	Brain	Dead	4
12	M	59	p.V600E	Lymph nodes	M1a	CR (SR)	N	NA	Alive	3
13	F	42	p.V600E	Brain, peritoneum, lymph nodes, liver, soft tissues, lung, adrenal gland	M1d	PR	Y	Brain	Dead	4^c^
15	F	46	p.V600E^b^	Lung, bones	M1c	PR (SR)	N	NA	Alive	3
17	M	48	p.V600E	Soft tissues, thoracic wall, bones, subcutaneous, abdominal wall	M1c	CR	Y	Bones, soft tissues, peritoneum, lung	Dead	2
19	M	64	p.V600E	Lung, lymph nodes	M1b	PD	Y	Brain, lung, spleen, lymph nodes	Dead	2
24	M	51	p.V600E^b^	Tonsils, lymph nodes	M1c	CR (SR)	N	NA	Alive	3
27	M	85	p.V600K	Lymph nodes, liver	M1c	PR	N	Liver, lung, adrenal gland, spleen	Dead	4
28	M	84	p.V600E	Subcutaneous, lymph nodes, liver, bones	M1c	PR	N	Adrenal gland, brain	Dead	3
34	F	75	p.V600K^b^	Lung	M1b	PD	Y	Peritoneum, lung	Dead	1
48	M	59	p.T599dup	Pleura, lung, lymph nodes	M1b	PR	N	Pleura, liver, lymph nodes, brain	Alive	3
49	M	64	p.V600E	Brain, liver, lung, lymph nodes, bones	M1d	PD	Y	Brain	Dead	1

*SD* stable disease, *PR* partial response, *CR* complete response, *SR* still responding, *PD* progressive disease, *EP* early progression, i.e. progression free survival ≤6 months, *Y* yes, *N* no, NA not appropriate, *Number of samples* total number of plasma samples collected for every patient.

^a^BRAF mutation identified in the liquid biopsy if not otherwise indicated.

^b^BRAF mutation identified in the FFPE tissue sample.

^c^Additional samples corresponding to therapy change after progression and one month later were analyzed^46^.

**Table 2:** Liquid biopsy quantitative details of the 17-patient cohort

Patient ID	BRAF mutant ctDNA copies^a^				pTERT mutant ctDNA copies^b^				CMC^c^	γH2AX + CMC^c^	CMC^c^	γH2AX + CMC^c^	CMC^c^	γH2AX + CMC^c^	CMC^c^	γH2AX + CMC^c^
	T0	T1	T2	P	T0	T1	T2	P	T0		T1		T2		P	
1	7.6	UD	NA	8.6	UD	ND	NA	UD	0	0	0	0	NA	NA	0	0
3	13768.3	17.4	ND	4365.2	UD	UD	ND	UD	5	1	1	1	ND	ND	1	0
6	1.7^d^	UD	UD	42.5	UD	UD	UD	5.11^d^	1	0	1	0	0	0	0	0
8	1190.1	UD	UD	ND	91.7	ND	UD	ND	0	0	0	0	0	0	ND	ND
9	128.2	UD	3	74.2	40.7	UD	3.6	29.3	12	1	0	0	0	0	3	1
10	UD	UD	UD	UD	UD	UD	UD	UD	0	0	0	0	0	0	0	0
12	167.7	UD	UD	NA	32.7	ND	UD	NA	0	0	5	0	0	0	NA	NA
13	10432.1	NA	NA	8469.4	7917.5	NA	NA	684.2	5	3	NA	NA	NA	NA	1	1
15	UD	UD	UD	NA	UD	ND	UD	NA	0	0	0	0	0	0	NA	NA
17	399.4	NA	NA	UD	146.1	NA	NA	UD	1	0	NA	NA	NA	NA	0	0
19	694.4	NA	NA	2241.1	191.2	NA	NA	340.8	2	0	NA	NA	NA	NA	0	0
24	UD	UD	UD	NA	UD	ND	UD	NA	0	0	0	0	1	0	NA	NA
27	99.9	791.4	UD	48633.1	20.2^d^	152.0	UD	934.3	0	0	0	0	1	1	0	0
28	4711.5	UD	NA	141008.7	806.0	UD	NA	233.9	9	3	0	0	NA	NA	1	1
34	2.1^d^	NA	NA	ND	3.2^d^	NA	NA	ND	2	2	NA	NA	NA	NA	ND	ND
48	17.8	UD	ND	5.54	8.9^d^	UD	UD	5.5^d^	failed	failed	0	0	NA	NA	1	1
49	2673.9	NA	NA	ND	UD	NA	NA	ND	1	1	NA	NA	NA	NA	ND	ND

*UD* undetectable, *NA* not appropriate, *ND* not done, *T0* baseline, *T1* six months follow-up, *T2* ten months follow-up, *P* progression.

^a^BRAF mutant copies per ml of plasma.

^b^pTERT mutant copies per ml of plasma.

^c^CMC per 7.5 ml of blood.

^d^Below quantitative limit (BQL). Undetectable: < LOD (0.06–0.5% for BRAF and 0.04–1.1% for TERT depending on sample concentration and availability for multiple replicates).

### Longitudinal assessment of BRAF-mutant ctDNA identifies a correlation with response to treatment

The clinical utility of the timing of BRAF-mutant ctDNA tracking was investigated in all 50 plasma samples (Table 2). Once routinely applied, this technique revealed a turnaround time of one day, or two days including cfDNA extraction.

**Fig. 2:**
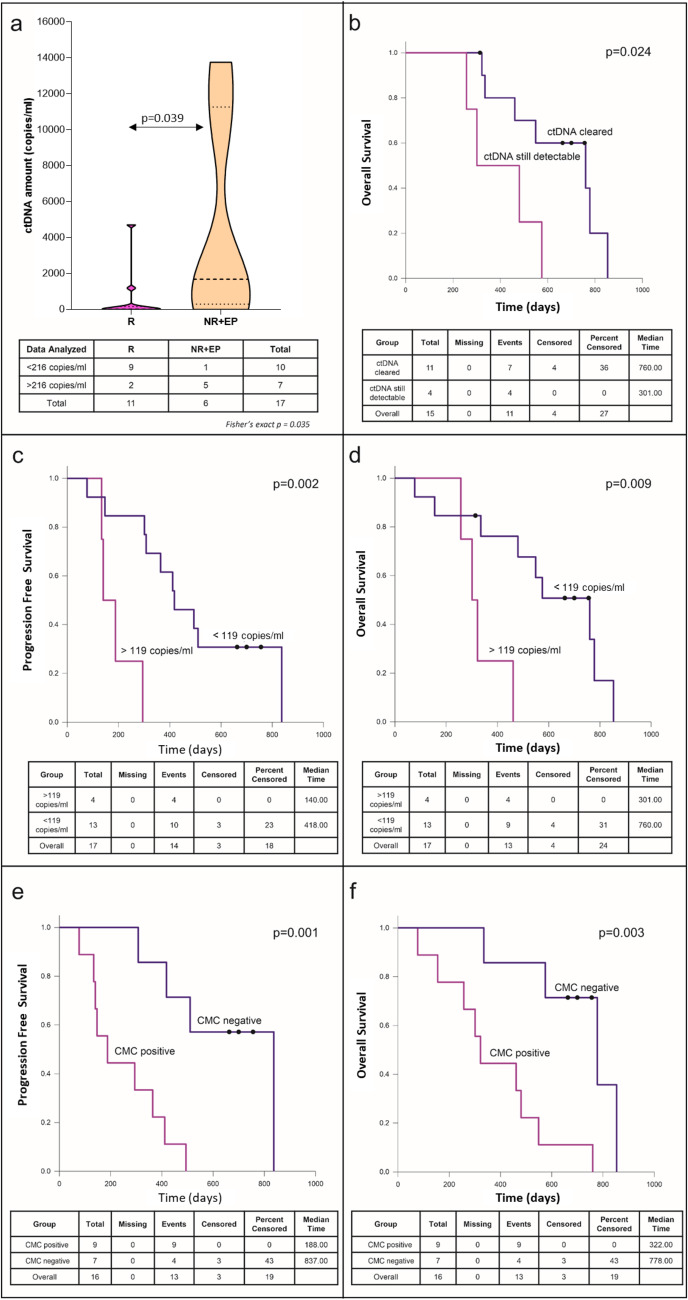
Correlation of ctDNA and CMC with response to treatment, PFS and OS. **a** Violin plot illustrating the amount of ctDNA (copies/ml of plasma) at T0 in responders vs non-responders/early progressing patients (Mann–Whitney *U* test, *p* = 0.039). First and third quartile, together with the median (middle line) are indicated in the plot. Contingency table with corresponding Fisher’s exact test *p* value is indicated below the graph. R, responding; NR, not responding; EP, early progressing patients (PFS ≤ 6 months). Kaplan–Meier plots of (**b**) OS according to BRAF-mutant ctDNA clearance at the first observational point after treatment start (range 2–6 months); (**c**) PFS and (**d**) OS according to baseline pTERT-mutant ctDNA amount; (**e**) PFS and (**f**) OS according to CMC count at baseline. The violin plot was performed using GraphPad version 8.0 for Windows (GraphPad Software Inc., San Diego, CA, USA).

**Fig. 3:**
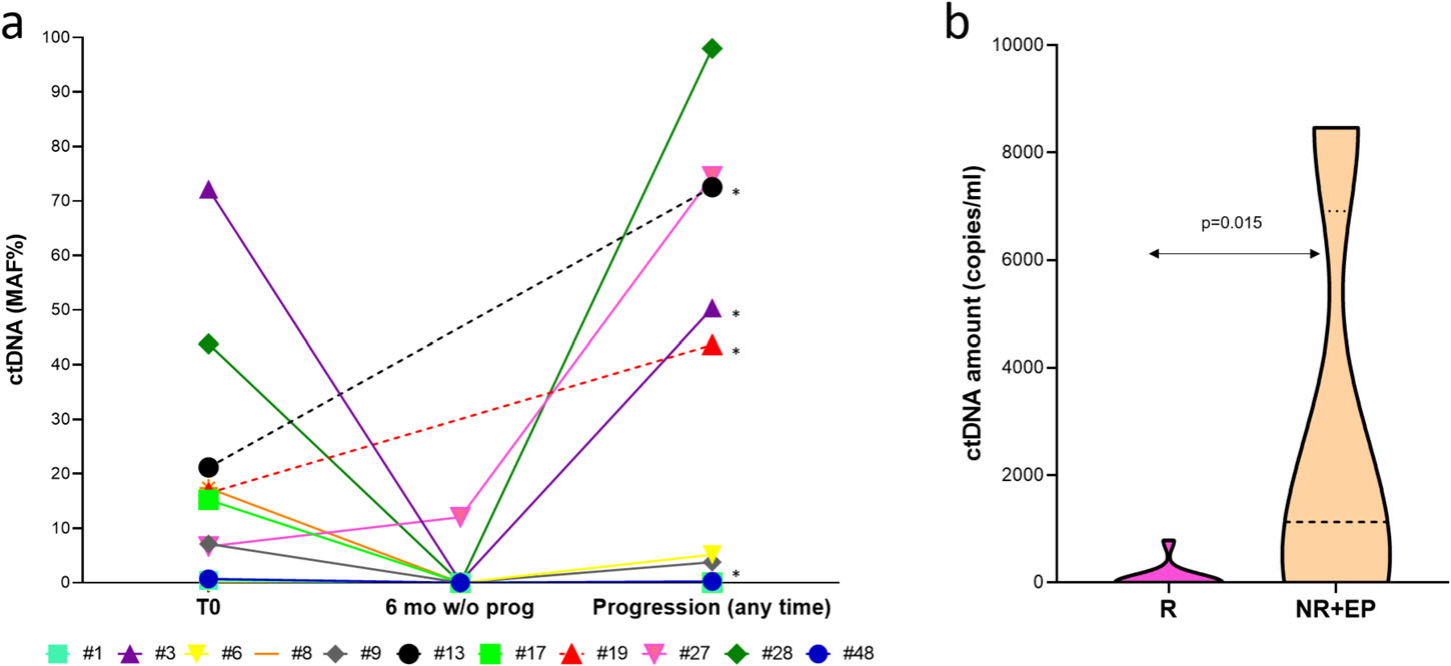
Longitudinal ctDNA dynamics and correlation between lack of clearance in the first 6 months and response to treatment. **a** Longitudinal monitoring of BRAF ctDNA MAF, shown for every patient, and (**b**) violin plot of BRAF-mutant ctDNA amount (copies/ml of plasma) evaluated at the first observational point after the beginning of therapy (range 2–6 months). ctDNA amount is significantly different between responders and non-responders/early progressing patients (Mann–Whitney *U* test *p* = 0.015). T0, baseline; 6 mo w/o prog: 6-month follow-up without antecedent progression; *:not responding and/or early progressing patients; MAF, mutant allele fraction; R, responding; NR, not responding; EP, early progressing patients (progressed before 6 months of treatment). The graph and the violin plot were performed using GraphPad version 8.0 for Windows (GraphPad Software Inc., San Diego, CA, USA).

**Fig. 4:**
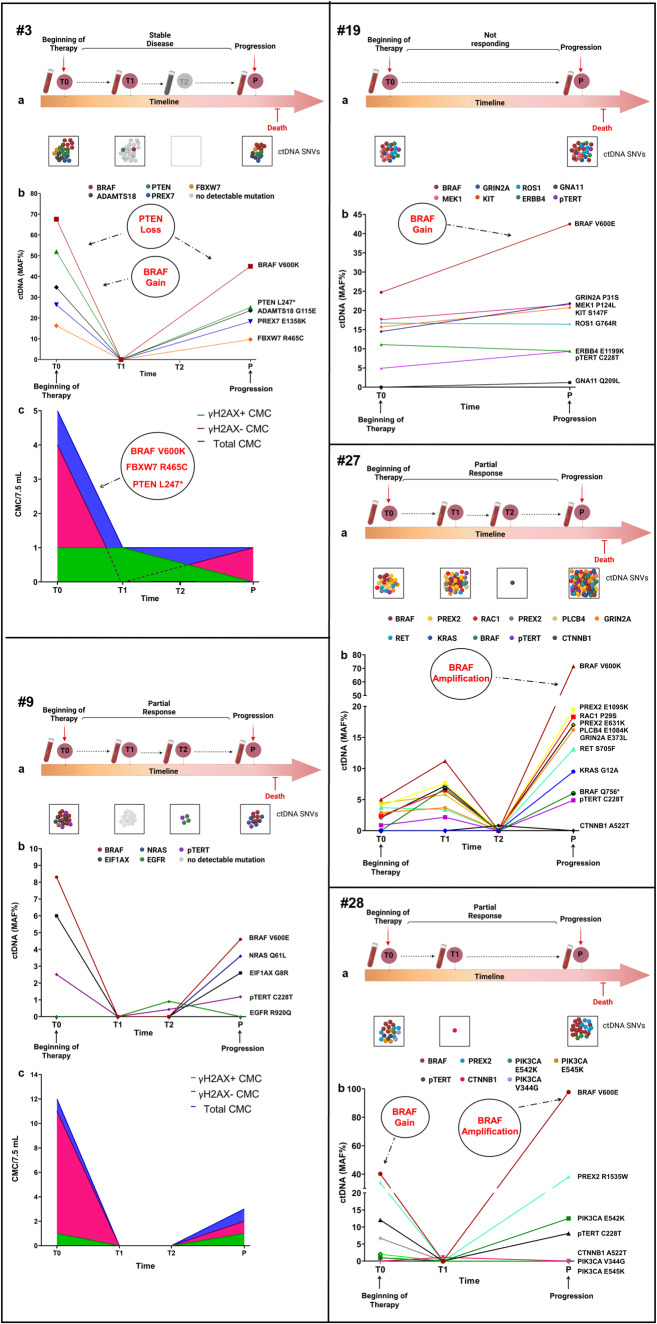
Liquid biopsy dynamics. Timeline (**a**) and longitudinal plot (**b**) of SNV MAFs detected in ctDNA of patients #3, #9, #19, #27, #28. CMC count dynamics (**c**) is shown for patients #3 and #9. The timeline was created with BioRender (https://biorender.com/); the plot was performed using GraphPad version 8.0 for Windows (GraphPad Software Inc., San Diego, CA, USA).

The BRAF-mutant fraction ranged from 0.02% to 98% and was detected at baseline in 82% of the patients (14/17, Table 2), in line with what already reported^39^ (Supplementary Table 1). The number of BRAF-mutant ctDNA copies/ml in plasma varied among samples from 1.7 to 141,008.7 (Table 2), and the amount of ctDNA (copies/ml of plasma) at T0 was significantly different between responders and non-responders/early progressing patients (Mann-Whitney U Test, *p* = 0.039; Fig. 2a). Therefore, we evaluated the potential of the baseline BRAF-mutant ctDNA amount in predicting the type of response or early progression (≤6 months), and identified a cut-off of 216 copies/ml of plasma (Fig. 2a and Supplementary Fig. 2). This value discriminated between complete/partial responders and stable/progressing patients. Most importantly, this cut-off also discriminated early progressing patients regardless of the type of response (Fisher’s exact test *p* = 0.035). Moreover, we considered on-treatment changes to assess whether BRAF-mutant ctDNA dynamics could have a clinical impact, in addition to the baseline evaluation. Treatment induced a reduction in the number of mutant copies; indeed, the amount of ctDNA decreased up to becoming undetectable in most patients at the first observational point (corresponding to T1 in the case of protracted response, or to progression, if earlier) (Table 2). Interestingly, undetectable BRAF-mutant ctDNA at that timepoint correlated with OS (Kaplan–Meier Survival Analysis, log-rank test, *p* = 0.024, Fig. 2b) and type of response. In this regard, the lack of BRAF-mutant ctDNA clearance up to the first 6 months of treatment correlated significantly with NR or early progression, suggesting a further endpoint for ctDNA as a biomarker (Mann–Whitney *U* Test, *p* = 0.015; Fig. 3). Finally, among patients with detectable BRAF-mutant ctDNA at baseline and a further observational point (Table 2), we observed ctDNA rebound in 9/10 patients upon progression (Supplementary Table 1, Fig. 3, and Fig. 4), mirroring the rise of resistance (intrinsic or acquired) and the consequent evasion from BRAFi/MEKi control^25,26,40,41^.

### pTERT-mutant ctDNA is informative but underrepresented if compared to BRAF-mutant counterpart

We tested the entire cohort of 17 metastatic melanoma patients for C228T and C250T TERT promoter (pTERT) mutations by ddPCR, as this region was not covered by the NGS panel. Fifty-nine% (10/17) and 41% (7/17) of the patients had a detectable mutation at baseline and progression, respectively. The number of pTERT C228T/C250T ctDNA copies/ml of plasma varied among samples from 3.2 to 7917.5 (Table 2). pTERT-mutant ctDNA was underrepresented compared to both BRAF-mutant ctDNA and several single nucleotide variants (SNVs) detected by NGS (Table 2, Supplementary Table 1 and Fig. 4). Nonetheless, we identified a cut-off for ctDNA amount at baseline that allowed the discrimination of patient outcome before the beginning of therapy. Indeed, patients with > 119 pTERT-mutant copies/ml had a worse progression-free survival (PFS, median 418 days vs. 140 days, *p* = 0.002) and OS (median 760 vs. 301 days, *p* = 0.009) (Fig. 2c, d).

### Validating the custom NGS panel

Supplementary Table 2 summarizes and compares the sequencing QC metrics of the 50 samples belonging to the melanoma patient cohort. For samples with higher DNA inputs (25–62 ng), a median percentage of on-target reads of 70% was obtained. Moreover, as coverage is a function of DNA input, the median depth was 2289 when starting from 25 to 62 ng and 1150 when starting from 10 to 24 ng. Finally, the percentage of duplicates was quite high (65%); nevertheless, it was in line with previous findings, as the duplication rate tends to be much higher for fragmented DNA of low quality, that is FFPE and cfDNA^42^. For samples below the threshold suggested by the manufacturer (cfDNA amount <10 ng), the QC metrics were less satisfactory. Nonetheless, we were able to trace tumor evolution in all samples.

The sensitivity and specificity of the SureSelect All-in-One custom panel, measured by means of the reference standard, were 96% and 100%, respectively, for mutant allele fractions (MAFs) up to 1% (PPV:100%, NPV:89%). The percentage reached 100% for both parameters for MAFs up to 5% (PPV:100%, NPV:100%) (Supplementary Table 3).

For mutations detected in cfDNA from patient samples and validated using ddPCR analysis, MAFs almost overlapped (Supplementary Table 4). Specifically, SNVs were correctly detected down to 0.8% (set as the limit of detection) in 100% of cases (43/43 SNVs). The panel performed well for MAFs down to 0.2%, with 5 out of 6 SNVs detected from the BAM file inspection. Since there was not enough cfDNA to run technical replicates, we set the threshold for variant calling at 0.8%, as this threshold was supported by data from the analysis of the certified control. We reserved the right to call mutations with MAFs lower than 0.8% only for specific cases, where the presence of the mutation was supported by other evidence and/or orthogonal techniques^43^.

During the validation process, we excluded false positive calls based on the presence of strand bias or because of the existence of read alignments with a pseudogene, that is, in the case of GNAQ p.M59L, p.T96S, and p.Y101* (19/50 samples), at a MAF of approximately 1% (BAM file analysis)^44^. Moreover, we detected PIK3CA p.R524K (MAF of 1%) owing to misalignment with the PIK3CA-pseudogene located on chromosome 22^45^. We also excluded TP53 p.R175H, detected at a MAF of 1.8-3.3% in the cfDNA samples of patient #27, because we identified the same SNV with a similar MAF (1.8-3%) on matched DNA from white blood cells (WBC), a feature that unveils a hematopoietic origin (Supplementary Table 5).

For copy number assessment, we tested the concordance between the BRAF copy number defined by ddPCR and the data obtained from the SureSelect All-In-One custom panel. We determined that a copy number indication of 3 was a real gain only if detected by all probes covering the gene; otherwise, the copy number should be considered normal, as confirmed by ddPCR. A 100% sensitivity and 97.5% specificity were reached, increasing to 100% for copy number variation (CNV) above 5 (Supplementary Table 6).

### cfDNA profiling and SNV load associate with resistance

To investigate the utility of ctDNA as a surveillance biomarker in melanoma patients undergoing targeted therapy, patients were longitudinally monitored via NGS (turnaround time of approximately 15 days) to detect multiple mutations suggestive of resistance and/or progression. This longitudinal monitoring allowed us to map the alterations present already at the baseline (T0) and those later at progression (P), which are likely responsible for intrinsic or acquired resistance, respectively.

The NGS panel was used to analyze 52 clinically relevant genes covering, among others, melanoma drivers^46^. The cfDNA analysis is summarized in Table 3, where somatic SNVs with a COSMIC ID (https://cancer.sanger.ac.uk/cosmic)^47^ are reported divided per patient and specific time points. cfDNA SNVs were detected in 76% (13/17) of patients at MAFs ranging from 0.98% to 67.6% at baseline, and from 0.8% to 97.7% at progression. To determine whether ctDNA would be a good surrogate for tracking tumor heterogeneity and to confirm that somatic cfDNA SNVs were tumor-derived, custom panel sequencing was performed on matched tumor tissue DNA when available. Tumor tissue/cfDNA concordance ranged from 81% to 100% for SNVs at MAF > 0.8% or > 1.5%, respectively (Supplementary Table 7).

**Table 3:** cfDNA somatic SNVs detected longitudinally by NGS analysis

Patient ID	Gene	Chromosomal location	Position (hg38)	nt change	AA change	Type of change	COSMIC ID	cfDNA amount (MAF %)			
								T0	T1	T2	P
1	BRAF	7q34	140753336	T > A	V600E	Nonsynonymous	COSM476	1.1	UD	ND	UD
3	ADAMTS18	16q23.1	77364300	G > A	G115E	Nonsynonymous	COSM142238	34.8	UD	ND	23.6
	BRAF	7q34	140753336-140753337	DelinsAA	V600K	Nonsynonymous	COSM473	67.6	UD	ND	44.9
	FBXW7	4q31.3	152328233	C > T	R465C^a^	Nonsynonymous	COSM22932	16.3	UD	ND	9.7
	PREX2	8q13.2	68138502	G > A	E1358K	Nonsynonymous	COSM3650517	26.4	UD	ND	18.3
	PTEN	10q23.31	87957958	T > G	L247*^a^	Stopgain	COSM3736942	52.0	UD	ND	25.1
6	ABL1	9q34.12	130854948	G > A	R153H	Nonsynonymous	COSM6934804	1.1	UD	UD	UD
	BRAF	7q34	140753336	T > A	V600E	Nonsynonymous	COSM476	UD	UD	UD	6.6
	RET	10q11.21	43124939	C > T	A999V	Nonsynonymous	COSM6240510	1.1	UD	UD	UD
	SF3B1	2q33.1	197409962	C > T	R238C^b^	Nonsynonymous	COSM4506189	UD	UD	UD	1.3
	SF3B1	2q33.1	197418534	G > A	R157Q	Nonsynonymous	COSM4983074	1.3	UD	UD	UD
8	BRAF	7q34	140753336	T > A	V600E	Nonsynonymous	COSM476	17.1	UD	UD	ND
	CTNNB1	3p22.1	41224633	A > G	T41A	Nonsynonymous	COSM5664	6.3	UD	UD	ND
9	BRAF	7q34	140753336	T > A	V600E	Nonsynonymous	COSM476	8.3	UD	UD	4.6
	EGFR	7p11.2	55200385	G > A	R920Q	Nonsynonymous	COSM7338932	UD	UD	0.9	UD
	EIF1AX	Xp22.12	20138617	G > A	G8R	Nonsynonymous	COSM3372215	6.0	UD	UD	2.6
	NRAS	1p13.2	114713908	A > T	Q61L^b^	Nonsynonymous	COSM583	UD	UD	UD	3.6
12	ARID2	12q12	45850473	C > T	P784S	Nonsynonymous	COSM5413317	14.7	UD	UD	NA
	BRAF	7q34	140753336	T > A	V600E	Nonsynonymous	COSM476	18.2	UD	UD	NA
13	ATM	11q22.3	108330374	C > T	L2490F	Nonsynonymous	COSM327924	26.1	NA	NA	35.5
	BRAF	7q34	140753336	T > A	V600E	Nonsynonymous	COSM476	20.6	NA	NA	73.1
	MEK1	15q22.31	66436825	C > T	P124L^a^	Nonsynonymous	COSM1315861	23.7	NA	NA	27.7
17	BRAF	7q34	140753336	T > A	V600E	Nonsynonymous	COSM476	25.7	NA	NA	UD
	PLCB4	20p12.3-p12.2	9338047	C > T	R69W	Nonsynonymous	COSM2933898	31.8	NA	NA	UD
19	BRAF	7q34	140753336	T > A	V600E	Nonsynonymous	COSM476	24.7	NA	NA	42.5
	ERBB4	2q34	211383947	G > A	E1199K	Nonsynonymous	COSM4764537	11.1	NA	NA	9.4
	GNA11	19p13.3	3118944	A > T	Q209L^b^	Nonsynonymous	COSM52969	UD	NA	NA	1.2
	GRIN2A	16p13.2	10180321	C > T	P31S	Nonsynonymous	COSM2141702	14.5	NA	NA	21.8
	KIT	4q12	54698386	C > T	S147F	Nonsynonymous	COSM5904789	15.7	NA	NA	20.7
	MEK1	15q22.31	66436825	C > T	P124L^a^	Nonsynonymous	COSM1315861	17.6	NA	NA	21.5
	ROS1	6q22.1	117385697	G > A	G764R	Nonsynonymous	COSM3157846	16.7	NA	NA	16.4
27	BRAF	7q34	140734632	C > T	Q756*	Stopgain	COSM9746164	UD	6.8	UD	6.0
	BRAF	7q34	140753336-140753337	DelinsAA	V600K	Nonsynonymous	COSM473	5.0	11.2	UD	71.5
	CTNNB1	3p22.1	41234178	G > A	A522T	Nonsynonymous	COSM3408665	UD	UD	0.8	UD
	GRIN2A	16p13.2	9890991	G > A	E373K	Nonsynonymous	COSM6918809	2.9	3.7	UD	16.2
	KRAS	12p12.1	25245350	G > C	G12A^b^	Nonsynonymous	COSM522	UD	UD	UD	9.5
	PLCB4	20p12.3-p12.2	9468572	G > A	E1084K	Nonsynonymous	COSM3549595	4.4	5.8	UD	17.0
	PREX2	8q13.2	68083252	G > A	E631K	Nonsynonymous	COSM9876557	2.1	7.2	UD	17.0
	PREX2	8q13.2	68115889	G > A	E1095K	Nonsynonymous	COSM3650510	4.0	7.6	UD	19.4
	RAC1	7p22.1	6387261	C > T	P29S^a^	Nonsynonymous	COSM125734	2.4	6.5	UD	18.3
	RET	10q11.21	43114714	C > T	S705F	Nonsynonymous	COSM6932233	3.7	3.4	UD	13.1
28	BRAF	7q34	140753336	T > A	V600E	Nonsynonymous	COSM476	40.2	UD	NA	97.7
	CTNNB1	3p22.1	41234178	G > A	A522T	Nonsynonymous	COSM3408665	UD	1.1	NA	UD
	PIK3CA	3q26.32	179203761	T > G	V344G	Nonsynonymous	COSM22540	2.0	UD	NA	UD
	PIK3CA	3q26.32	179218294	G > A	E542K	Nonsynonymous	COSM760	1.0	UD	NA	12.5
	PIK3CA	3q26.32	179218303	G > A	E545K	Nonsynonymous	COSM763	6.7	UD	NA	UD
	PREX2	8q13.2	68192524	C > T	R1535W	Nonsynonymous	COSM340299	33.7	UD	NA	37.8
48	ERBB4	2q34	211702093	G > A	A455T	Nonsynonymous	COSM7344043	5.1	2.2	NA	2.3
	PPP6C	9q33.3	125158280	C > T	H151Y^b^	Nonsynonymous	COSM23144	UD	UD	NA	0.8
	RET	10q11.21	43124939	C > T	A999V	Nonsynonymous	COSM6240510	1.1	UD	NA	UD
49	BRAF	7q34	140753336	T > A	V600E	Nonsynonymous	COSM476	41.9	NA	NA	ND
	MET	7q31	116769777	G > A	E906K	Nonsynonymous	COSM5576816	20.0	NA	NA	ND
	TP53	17p13.1	7674241	C > T	S241F	Nonsynonymous	COSM10812	44.5	NA	NA	ND

*AA* amino acid, *cfDNA* cell-free DNA, *COSMIC* Catalogue of Somatic Mutations in Cancer database, *MAF* mutant allele fraction, *NA* not appropriate, *ND* not done, *UD* undetectable, *nt* nucleotide. *Time points of blood collection during monitoring: T0* before starting the therapy, *T1 and T2* follow-up during clinically disease-free period, *P* progression.

^a^Mutations conferring intrinsic resistance.

^b^Mutations rising at the time of progression.

**Fig. 5:**
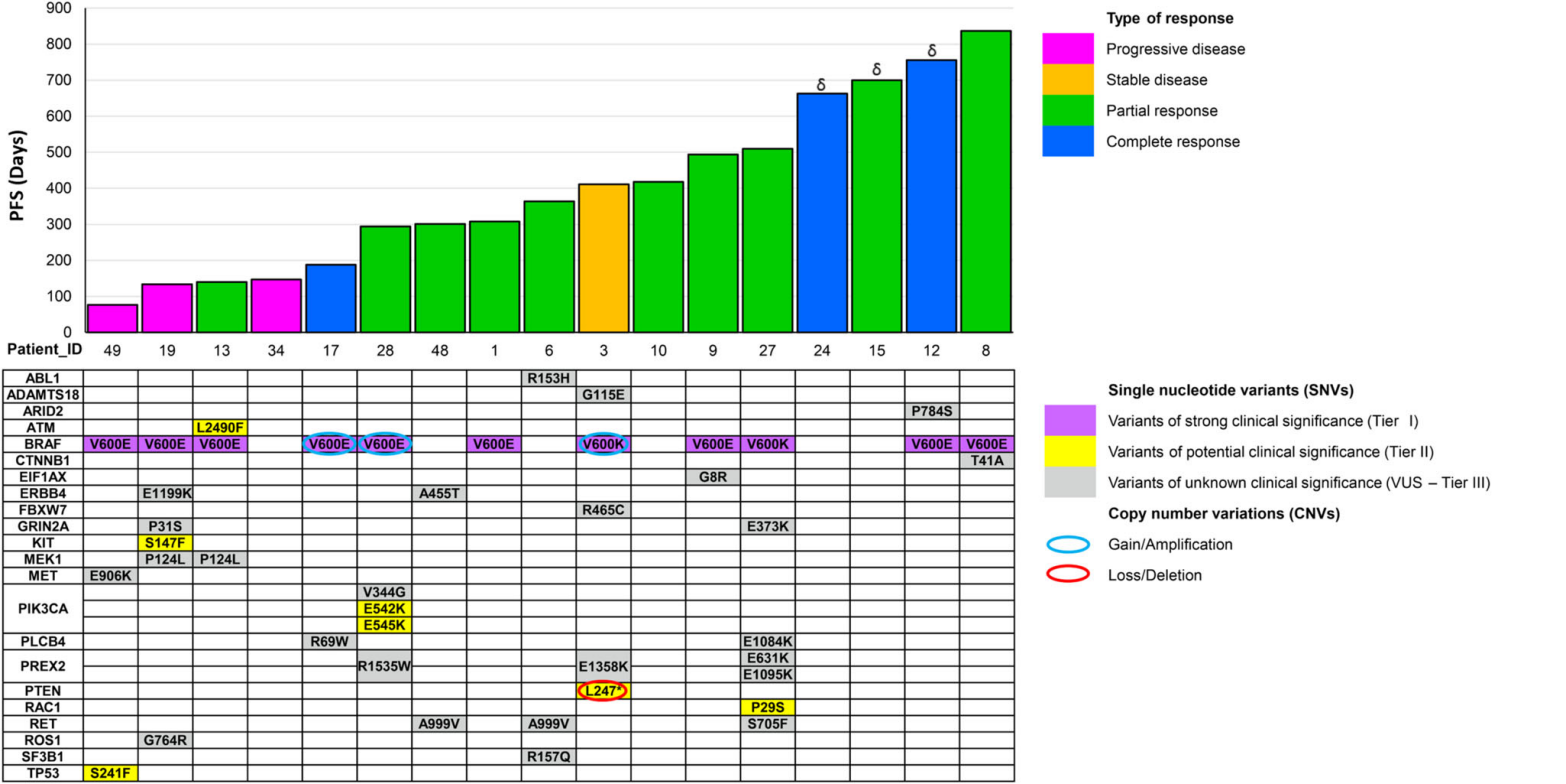
Summary of SNVs and CNVs detected by NGS in cfDNA at the baseline and type of response. Patients are sorted by PFS (days). ^δ^Patients still responding when data were collected. SNVs are classified according to Association for Molecular Pathology (AMP) guidelines. AMP classifications were obtained from Franklin website (https://franklin.genoox.com - Franklin by Genoox).

A representative cfDNA profile of some patients is reported in Fig. 4, together with the information gathered from the other analyses (ddPCR, CMC analysis, see the following paragraphs). As already shown, BRAF MAF, if detectable at baseline, mostly decreased during response and increased again at progression. More specifically, this parameter cleared in case of response or remained detectable in patients not responding to therapy. When two or more SNVs were detected at different time points, the ctDNA dynamics of these variants showed an overlapping or parallel trend consistent with clinical response (Fig. 4 and Table 3), regardless of whether they were melanoma drivers (BRAF p.V600E/K, NRAS p.Q61L, RAC1 p.P29S, PTEN p.L247*) or variants of uncertain significance (ADAMTS18 p.G115E, ATM p.L2490F, E1F1AX p.G8R, and KIT p.S147F)^36^. An increasing number of SNVs was associated with disease progression (matched sample *t* test, *p* = 0.036; Supplementary Fig. 3)^48^. Moreover, the number of different cfDNA SNVs (SNV load) at baseline correlated with treatment outcome, as patients with SNVs > 2 had a worse PFS (*p* = 0.041 Log rank test, Supplementary Fig. 3)^48^. These analyses highlight the ability of cfDNA to track residual or progressive disease (Fig. 4). Moreover, new SNVs that increased upon recurrence or mutations putatively able to confer resistance were observed in 50% (5/10) and 70% (7/10) of the patients, respectively (Table 3). This suggests a role for ctDNA as a pharmacodynamic marker in this combined approach, which is able to detect the presence and dynamics of different SNVs. Although fluctuating with the same trend as BRAF-mutant ctDNA, different resistance-responsible mutations were detected at different MAFs (Fig. 4, Table 3). Ruling out cases attributable to BRAF amplification, the remaining differences in MAF abundance were suggestive of the presence of different subclones. Patient #27, indeed, had multiple recognized melanoma drivers including BRAF p.V600K, RAC1 p.P29S, pTERT C228T and KRAS p.G12A, this latter arising at the time of progression; thus, patient #27 represents a good example of a combined intrinsic (RAC1 mutation) and acquired (KRAS mutation) resistance. The MAF increased in the plasma at month 6 (T1), decreased at T2, and then rebounded at progression. At this latter time point, the BRAF p.V600K amount was 4-fold higher than that of RAC1 p.P29S (at baseline, there was a 2-fold difference) and 7.5-fold higher than that of KRAS p.G12A, suggesting a gain in copy number that was confirmed by CNV assessment. This is a common mechanism of resistance to BRAF inhibitors^49^ together with the presence of different subclones. Patient #3 had multiple driver mutations (BRAF p.V600K, PTEN p.L247*, and FBXW7 p.R465C) that decreased during the stable disease interval and rebounded at the time of disease progression. CNVs in BRAF (gain) and PTEN (loss) genes were identified in parallel (Fig. 4), thus accounting for the lack of response to targeted therapy. Notably, patients #13 and #19 showed a similar timing of progression, and both carried the MEK1 p.P124L mutation that confers resistance to the MEK inhibitor trametinib^31,50^, and a BRAF gain as a combined strategy to overcome both the checkpoints of the targeted therapy^46^. Finally, patients who did not experience ctDNA clearance over the first 6 months (patients #3, #13, #19, # 27) turned out to carry a mutation conferring intrinsic resistance from the beginning (Table 2, Table 3, and Fig. 5).

### CMCs are a prognostic biomarker

Using the FDA-cleared CellSearch system, we detected CMCs in 56% (9/16) and 45% (5/11) of the patients at baseline and progression, respectively. The turnaround time for detection and enumeration of CMCs is normally 2 days. As previously reported^14,15^, CMCs confirmed to be a prognostic biomarker. Indeed, univariate analysis showed that 1 or more CMCs at baseline correlated with early progression (Fisher’s exact test, *p* = 0.034), worse PFS (Kaplan–Meier Survival Analysis, log-rank test *p* = 0.001; Fig. 2e) and OS (Kaplan–Meier Survival Analysis, log-rank test *p* = 0.003; Fig. 2f). Thus, our results confirm previous findings made in larger cohorts, among which one comprising stage IV melanoma patients and therefore highly related with our study^51,52^. On the other hand, CMCs failed as a predictive marker of response (CMC ≥ 2, Fisher’s exact test, *p* = 0.118).

The damaged γH2AX-positive fraction of CMCs (Supplementary Fig. 4) has been assumed as a surrogate for drug sensitivity and response to therapy^53,54^. However, we did not find any significant correlation between CMC status and clinical outcome, apart from some suggestive cases (i.e., patients #3 and #9, Fig. 4) where CMCs appeared mostly damaged during response/stability but viable (i.e., γH2AX-negative) at the time of progression, thus mirroring an actively proliferating disease^46^.

Next, we wondered whether the assessment of the genetic landscape of CMCs could provide information other than their absolute number. To this end, we firstly validated a workflow (Supplementary Fig. 5) encompassing upstream single-cell isolation and downstream molecular analysis of well-known tumor cell lines carrying traceable mutations, which underwent staining with different procedures. Supplementary Table 8 shows the results from 44 samples containing microdissected cells, either single or in clusters. Overall, whole genome amplification (WGA) was successful in 70% of cases, and mutations were correctly identified by ddPCR in 90% of cases, presenting an allelic imbalance (AI) or exclusion of one of the two alleles (allele dropout, AD) in 36% and 32% of the cases, respectively. DNA amplification by ddPCR failed in 10% of cases, in line with previous reports^55^. Finally, amplicon-based targeted sequencing (Ampli1 OncoSeek Panel) detected known hotspot mutations with good accuracy ranging from 60 to 100% (Supplementary Table 9). Focal CNVs were also identified. Otherwise, the panel correctly found no somatic mutations or CNVs in WBCs.

Subsequently, the Ampli1 OncoSeek Panel was employed on 15 samples from 9 patients. Eleven samples showed very low uniformity and a substantial number of amplicons were covered by < 20 reads; therefore, they were excluded from the final analysis. Missense mutations were detected in 3/4 of the remaining samples from 2 different patients (Supplementary Table 10). The analysis revealed the presence of different mutations, some overlapping with those detected in the ctDNA counterpart. At T0, patient #3 showed the presence of BRAF p.V600K (MAF 99.82%), FBXW7 p.R465C (MAF 31.39%), and PTEN p.L247* (MAF 99.13%). The same mutations were detected in the corresponding ctDNA samples using the SureSelect All-In-One custom panel, although with lower allele frequencies, likely because of ctDNA contamination by normal cfDNA (Supplementary Table 10). No significant somatic mutations or CNVs were detected in the microdissected WBCs. For patient #13, we analyzed a sample corresponding to the time of targeted therapy change after progression (T2) and an additional time point one month later (T3)^46^. Analysis of the microdissected CMCs highlighted the presence of BRAF p.V600E (MAF 23.6%), which was also confirmed in the corresponding ctDNA sample by the SureSelect All-In-One custom panel (MAF 63.9%), ERBB4 p.F266S (MAF 100%), and TP53 p.R156H (MAF 17.9%), which were not detected in the cfDNA.

## Discussion

The main purpose of this study was to set up and validate a multiparameter approach applicable to different components of the liquid biopsy compartment. Together with the original design of the customized ctDNA NGS panel, this work is innovative not because of the exploited technologies per se but rather because of their integrated use and, mostly, for the timing set for their application. The enrolled cohort of patients with stage IV melanoma, although small, provided an opportunity to postulate its clinical utility in a real-world setting, and the longitudinal monitoring allowed to confirm some previous findings based on single-test output.

Here, we further demonstrated the usefulness of both CMC count as a prognostic factor^14,15^ and ctDNA amount/trend in predicting the response to targeted therapy^30,39,40,56,57^, and suggest that this latter can be a valuable companion biomarker for melanoma patient management (Supplementary Fig. 6). On the other hand, we reported a combined approach of NGS and ddPCR assays applied to both ctDNA and CMCs biomarkers at every time point. In this regard, the NGS custom melanoma panel appeared to be well suited for ctDNA analysis and showed significant correlation with ddPCR (reaching 100% concordance for mutations down to 0.8% of frequency), with the additional benefit of allowing the parallel monitoring of driver and target genes together with the assessment of the BRAF CNV.

From a quantitative standpoint, the amount of ctDNA was an effective marker of response to treatment prior to the beginning of targeted therapy. Additionally, our approach suggests a further endpoint for this biomarker other than the concept that the initial amount of ctDNA can be directly correlated with patient outcome^26,27,58–60^. In fact, the assessment of on-treatment early dynamic changes in ctDNA amount allowed the identification of the proneness to early progression and non-response in patients without ctDNA clearance up to the first 6 months (Fig. 3). Even more interestingly, the same patients who did not clear their ctDNA amount at the first observational point were found to carry from the baseline a mutation known to confer intrinsic resistance to the treatment (Table 3). The parallel qualitative contribution provided by the NGS analysis supported the quantitative data obtained from ctDNA amount and trend, with the identification of mutations putatively conferring a specific advantage for tumor escape. In this regard, it has been hypothesized that the presence of a resistance mechanism might represent a confounding factor for the prognostic value of ctDNA^61^. Nonetheless, in our setting, we identified a strong correlation between the lack of ctDNA clearance and the type and duration of response, leading to assume the ctDNA trend as an early indicator of intrinsic resistance (Fig. 5, patients #3, #13, #19, #27, and Table 3). This envisions a synergistic use of ctDNA tracking and NGS analysis under the guidance of the BRAF-mutant ctDNA absolute amount, which appears more accurate in defining the tumor burden and more reliable in comparing different time points being unaffected by external influences. For this reason we decided to use this parameter in defining cut-offs and discriminating different categories. On the other hand, for the description of tumor heterogeneity in some plots and tables we used the MAF parameter, which is representative of the clonal composition of ctDNA and provides a qualitative overview of the patient’s mutational landscape. These parameters can be used alternatively because the two trends (absolute quantity and MAF) overlap for almost all the samples. Indeed, in this cohort and in our whole experience, we identified just a single case in which the trends of MAF and absolute amount were divergent^46^.

As BRAF-mutant ctDNA levels can be low in some patients, before and/or after the beginning of the targeted therapy, their tracking requires an extremely sensitive detection method. At the same time, for a putative clinical implementation, the same method should also guarantee short turnaround times together with low analysis costs. In our workflow, once being set routinely, ddPCR has shown a turnaround time of one day and a low-cost impact, confirming to be a good candidate to be implemented in the clinical setting. Moreover, NGS analysis can be completed within 15 days being thus compatible with the time frame of standard diagnostics. Additionally, the costs of NGS are not as prohibitive as they used to be when compared with those of imaging examinations or the costs of a therapy administered to a patient who cannot benefit from it. Finally and as suggested by our data, NGS should be performed only at a few and well-defined time points chosen following the outcome of the ctDNA test. In this regard, the proposed multiparametric approach naturally leads to integrate and weighing the different variables for their importance in predicting the patient outcome. In particular, the information related to the analysis of different quantitative and qualitative aspects of ctDNA appears the most suitable for a rapid translation into the clinics. On the other hand, the CMC count turned out less useful as a predictive marker, although being undoubtedly endowed with prognostic value. With the intent of suggesting a merely putative workflow that can help the optimal management of disease evolution, we selected the different variables considered of importance at different observational time points to build a flow chart (Supplementary Fig. 6).

Definitely, further trials are warranted to investigate whether patient surveillance using this approach can improve their clinical outcome. Whether we consider the results of this pilot study, it appears reasonable to develop a follow-up protocol that includes observational time points even earlier than those defined in the current work and closer to the start of the therapy, in order to identify more precociously those patients undergoing non-response and/or progression. Since a durable response to targeted therapy is only observed in a limited number of patients, whenever evidence suggests that the response will not last long, switching patients before the onset of recurrence to second-line immunotherapy or combo-immunotherapy will likely increase the chance of their success. Thus, this study provides the information required to define patients at major risk and offers a putative companion diagnostic tool for patients undergoing targeted therapy.

We also tracked the amount of pTERT mutant ctDNA to assess its potential as a good biomarker for disease monitoring. As already reported, melanoma patients harboring pTERT mutations in combination with BRAF/NRAS mutations have a significantly shorter PFS than patients without this combination^62,63^. Moreover, we additionally provided a cut-off to better stratify patients even from the baseline. Our results underlie the need for further studies prior to clinical implementation, as pTERT-mutant ctDNA was present in amounts lower than those of other tracked mutations and this can potentially impinge on liquid biopsy applications. This could be due to the low nucleosome occupancy of pTERT sites and the consequent lack of protection from nuclease cleavage^36,64^. Nonetheless, as mutations in pTERT enhance TERT expression, which in turn correlates with worse PFS particularly in melanoma^36,63^, the detection of a pTERT mutation optimized for low amounts may help in identifying patients at major risk of recurrence^65^.

Several pieces of evidence point in the direction of a polyclonal characteristic of acquired resistance^66^. Accordingly, several molecular alterations were concurrently detected in the samples from progressing patients (#3, #19, and #27), together with CNVs. Acquired mutations, not present prior to therapy initiation, were identified together with mutations detected from the baseline and putatively responsible for the incomplete or absent response to treatment (Table 3). This is an additional proof that cfDNA could accurately depict tumor heterogeneity and evolution, although some concerns arise in the presence of brain metastatic disease, as the blood-brain barrier could hamper the release of nucleic acids in the bloodstream. A study by Diefenbach et al. postulated that ctDNA is still representative of the systemic disease, likely because brain metastases from a genetic point of view have similar mutational and treatment response profiles of concurrent extra-cranial tumors^67^. In some of our patients with brain disease (#13, #19, #48), we could take advantage of at least one marker (ctDNA or CMCs) for the correct identification of progression and putative causative mutations; otherwise, in some other patients (#1, #8, #10), we did not detect signs of resistance.

Despite this limitation, pathogenic driver/causative mutations other than BRAF mutations have been identified at progression in 64% (7/11) of patients, thus showing that our customized NGS panel provided relevant information about the causative mutations for most patients. As targeted drugs have become mainstream in cancer treatment^68^ and new molecules are continuously being released, this type of information will be of increasing importance in the near future. In this regard, the management of patient #13 demonstrated that this workflow also has the potential to suggest second-line therapy when applied in real time^46^. A similar approach could have been set for patient #19 and also for patient #48^43^, as the mutation in the PPP6C gene found at progression in the latter is supposed to be sensitive to Aurora kinase inhibitors^69^. Moreover, the use of information from both ctDNA and CMC analyses represents the ideal situation (i.e., patients #3 and #9), as the two sources of information have to be considered complementary rather than overlapping^12^.

An issue in the analysis of plasma cfDNA is represented by clonal hematopoiesis (CH), a process involving the accumulation of somatic mutations in hematopoietic stem cells that leads to clonal expansion of mutated blood cells. CH is part of the normal aging process and is a common premalignant condition in the general population^70,71^. Mutations of hematopoietic origin represent a confounding factor in the interpretation of NGS data from plasma cfDNA, because they could be misclassified as tumor-derived mutations^72^. Since in our cohort the median age is 64 years, considerable attention was spent on the analysis of NGS data to avoid misinterpretation of these SNVs as cancer-associated variants. Paired NGS analysis of cfDNA samples and DNA extracted from blood cells represents the best approach for detecting CH^73^. However, the costs associated with paired sequencing is currently an impeding factor for the routinary use of this approach in clinical practice. Accordingly, we believe that a careful evaluation of the longitudinal MAF trend during patient follow-up, may enable the identification of CH-related mutations without the routinary screening of blood cells. In fact, CH-related mutations will show a low and constant MAF that is longitudinally stable. A subsequent ddPCR analysis on matched DNA extracted from blood cells will allow to rule out the tumor origin of the mutation in those selected cases that comply with the above characteristics at a more affordable cost.

All the evidence in favor of a strong heterogeneity of advanced melanoma leads to the consideration that the enrichment of CMCs based on a specific surface marker, as performed by the CellSearch platform, could be biased by the presence of subsets of tumor cells lacking that appropriate marker^5,74^. Thus, the CMCs we analyzed are most likely a subgroup of the entire CMC population. Conversely, we can state that ctDNA analysis could be representative of systemic disease and, at various grades, of all different subclones. Recent studies have demonstrated that a higher presence of specific mesenchymal antigens, such as CD146, is related to alterations in the MAPK pathways and/or an aggressive and higher metastatic phenotype^5,74^. Considering that CD146 is used by the CellSearch platform for the enrichment of the CMC fraction, it could be inferred that we analyzed the subpopulation with the higher potential to metastasize and putatively to acquire resistance. A tentative cut-off of 2 CMCs/7.5 ml blood was initially suggested as optimal in several studies, although the cut-off for melanoma patients is still under debate^14,15^. More recently, two studies performed on cohorts of stage III and IV melanoma patients showed that the presence of even one CMC was sufficient to confer a higher risk of early progression^51,52^. Accordingly, our data confirmed the prognostic potential of CMC count and the correlation of ≥1 CMC/7.5 ml blood with early progression, and worse PFS and OS. Conversely, CMCs failed as a predictive marker of response which is the ultimate goal.

We are aware that this study has some limitations. First, our cohort was small and encompassed only patients with stage IV melanoma, which could jeopardize the applicability of the results to wider settings. Nevertheless, from the perspective of patient age, the cohort was heterogeneous and not overtly shifted toward the older patients. Accordingly, we do not expect that tumor evolution has been influenced by age, and therefore we feel confident to assume that our data, although preliminary, can describe and characterize advanced melanoma in its heterogeneity. Second, the lack of closer observational time points for an early tumor assessment is one of the points that require implementation to strengthen the power of this biomarker. Third, the genetic analysis of CMCs showed a success rate of 27%, suggesting the presence of some hindrances both in the upstream recovery phase and in the downstream sequencing. Indeed, the whole process of CMC enrichment through CellSearch platform and CMC isolation by laser capture microdissection requires two fixation steps. Consequently, the effects exerted by fixative agents on chromatin accessibility could introduce some bias during whole genome amplification, because of the difficult amplification of genomic regions cross-linked to histone residues. Finally, the laser capture technique can potentially induce UV damage in the cells collected for analysis. Even with these limitations, this pilot setting proved that our liquid biopsy-based profiling of both cfDNA and CMCs allowed us to successfully track the evolution of the disease and to monitor the appearance of new druggable mutations.

In conclusion, we proposed a multiparameter, liquid biopsy-based approach that provides information to identify patients treated with BRAF/MEK inhibitors who are at major risk of recurrence and offers a potential companion diagnostic tool. This study assessed a longitudinal protocol for monitoring tumor genetic evolution through liquid biopsy analysis. The use of high-throughput methodologies optimized for low-quality DNA provided a reliable, real-time molecular picture of the tumor. Moreover, customized genetic profiling allows the identification of tumor vulnerabilities and dependencies, which can lead to the choice of different targeted/combined therapies. This work provides proof of principle of the power of this approach and paves the way for a validation study aimed at evaluating early ctDNA-guided treatment decisions in stage IV melanoma.

## Methods

### Patient samples and study design

Seventeen stage IV cutaneous melanoma patients carrying a BRAF mutation at amino acid position 600 (with the exception of patient #48 who carries the BRAF p.T599dup mutation^43^, Table 1), as identified in tissue biopsy, were prospectively enrolled and addressed to targeted therapy with BRAFi/MEKi. Patients were followed for up to 28 months (January 2019 to March 2022) to monitor disease evolution and to understand the timing/mechanisms of resistance. Considering that resistance develops at a median time of 11-12 months^4,75^, blood samples were collected just before starting therapy (T0), after 6 (T1), and after 10 (T2) months (to test the ability of the time setting to detect early signs of tumor escape), and at the time of progression (P). Approximately two to four serially collected blood samples were available per patient for a total of 50 samples (Table 1). Tumor response was determined by physical examination and imaging investigation using Response Evaluation Criteria in Solid Tumors (RECIST) v1.1^76^, and assessed between the first day of therapy until progression, death, or last follow-up: stable disease (SD), partial response (PR), complete response (CR), or progressive disease (PD). Responders (R) were defined as complete response or partial response. Non-responders (NR) were defined as stable disease or progressive disease. Decisions regarding treatment response or disease recurrence were made by the treating clinician who was blinded to the ctDNA results. All subjects involved in the study provided written informed consent.

The study was conducted in accordance with the guidelines of the Declaration of Helsinki and was approved by the Ethics Committee of the Veneto Institute of Oncology—IOV IRCCS (approval No. CESC-IOV 2018/36 on April 19, 2018).

### Sample collection, processing and storage

Blood samples were collected in different types of tubes following this order for each patient: CellSave tubes (Menarini Silicon Biosystems, Bologna, Italy) for CMC count, EDTA tubes, and Streck Cell-Free DNA BCT tubes (Streck, La Vista, NE, USA) for cfDNA analysis. Plasma was obtained by double centrifugation (1600 × *g* for 10 minutes and 16,000 × *g* for 10 min at room temperature) and stored at −80 °C. The cfDNA was isolated using the QIAamp Circulating Nucleic Acid Kit (Qiagen, Hilden, Germany), and quality control (QC) was performed using 4200 TapeStation system (cfDNA ScreenTape, Agilent Technologies, Santa Clara, CA, USA). QC was passed if the percentage of cfDNA was >82% (region table set from 50 to 700 bp, Supplementary Fig. 1). Samples for CMC enumeration were processed within 96 hours from blood collection.

### ddPCR analysis

Specific hotspots in BRAF gene and pTERT were assessed by ddPCR (BioRad Laboratories, Hercules, CA, USA), together with specific SNVs detected by NGS (Supplementary Material—Materials and Methods). Commercially available or customized probes were used according to the manufacturer’s instructions (Bio-Rad Laboratories). Specific conditions were applied for pTERT analysis (Supplementary Material—Materials and Methods, Table A). Positive (mutated DNA), negative (wild type DNA), and no-template (water) controls were included in each run. Data were acquired and analyzed using the QuantaSoft analysis software version 1.7.4 (BioRad Laboratories). Samples were defined as positive or positive below the quantitative limit (BQL) when ≥3, or 1-2 FAM-positive droplets were detected, respectively, with no positive droplets in the negative control. BRAF CNV was assessed by ddPCR using as references two probes located on chromosomes 14 and 7 (to discriminate BRAF gain from chromosome 7 polysomy)^77,78^. A probe on chromosome 10 was used to confirm PTEN loss in patient #3. To set the cut-off for defining gain and loss, 10 samples from healthy controls were analyzed. The cut-off was calculated as the mean copy number ± 1.96 SD (standard deviation), as already reported^79^. To evaluate technical reproducibility and/or increase the number of events, for those samples with a very low MAF, cfDNA was analyzed at least in duplicate, with most samples (48%) analyzed in triplicate/quadruplicate and 24% of samples analyzed in more than 4 replicates.

### NGS custom panel design and analytic evaluation

A hybridization capture-based target enrichment custom panel (SureSelect Cancer All-In-One custom panel, Agilent Technologies) was designed for the detection of SNVs and small deletions/insertions in 52 genes and CNV in 12 genes (Supplementary Material—Materials and Methods). The design covered hotspots for melanoma drivers, targetable mutations, and genes involved in pathways associated with treatment resistance, as described elsewhere^46^. Sequencing libraries were prepared using the SureSelect XT HS Target Enrichment System according to the manufacturer’s instructions (Agilent Technologies, Supplementary Material—Materials and Methods), and pools were sequenced on NextSeq 550 with 300-cycle NextSeq 500/550 Mid Output v2 kit (Illumina, San Diego, CA, USA). Alignment and variant calling were assessed using SureCall software v.4.2 (Agilent Technologies), with interpretation and prioritization by Alissa Interpret Analysis Software v.5.3.4 (Agilent Technologies).

The performance of the SureSelect Cancer All-In-One custom panel was evaluated by testing the correctness of the variant call with an orthogonal method (ddPCR system), and verifying the adherence of the MAF identified by NGS with a certified control (Multiplex I cfDNA Reference Standard Set 1% and 5%, Horizon Discovery, Cambridge, United Kingdom, 5 replicates, and WT control). ddPCR was used as an orthogonal method to assess both the presence and MAF of several SNVs identified by the sequencing of patient cfDNA, and the accuracy of the CNV identification.

### CMC capture and enrichment

CMCs were enriched from 7.5 ml peripheral blood samples through the CellSearch system using the Celltracks Circulating Melanoma Cell Kit (Menarini Silicon Biosystems) according to the manufacturer’s instructions. The DNA-damaged melanoma cells were identified by an integrated anti-γH2AX antibody (clone JBW301, Millipore Cat# 16-202A) that recognizes the phosphorylated form of histone H2AX (γH2AX), which is correlated to apoptotic chromatin fragmentation^53^. Results were expressed as the number of total and γH2AX-positive CMCs for 7.5 ml blood (Supplementary Material—Materials and Methods).

### Workflow validation from CMC isolation to NGS analysis

The CellSearch-enriched CMCs were picked up by laser capture microdissection with the MMI CellCut system (Molecular Machines & Industries GmbH, Eching, Germany) mounted on an ECLIPSE Ti2 microscope (Nikon Corporation, Tokyo, Japan), subjected to Ampli1 WGA (Menarini Silicon Biosystems) according to the manufacturer’s instructions (with minor modifications), and finally sent to Menarini Silicon Biosystems for NGS analysis with the Ampli1 OncoSeek Panel, which is designed specifically to fit with the Ampli1 WGA protocol (Supplementary Material—Materials and Methods). To validate the entire workflow, 44 samples from different tumor cell lines in the form of spike-in samples or cells suspended in their medium were enriched by the CellSearch platform or simply stained in culture (CellTracker Orange, Thermo Fisher Scientific) to undergo microdissection at the single-cell or cluster level, and finally WGA. Additional samples were also subjected to Ampli1 OncoSeek analysis to validate the entire workflow.

When analyzing patient samples, a minimum of 5 cells per CellSearch cartridge were microdissected to increase the chance of a successful WGA reaction. White blood cells were collected together if the number of CMCs was <5. The quality of the WGA products was tested using the Ampli1 QC Kit (Menarini Silicon Biosystems) following the manufacturer’s instructions.

### Statistics

The nonparametric Mann–Whitney *U* test was used for comparison between groups (ctDNA amount of responders vs. non-responders). A matched-sample *t* test was used to compare two sets of scores directly related to each other (SNV load before and after progression). Progression-free survival and overall survival were measured from the beginning of BRAFi/MEKi treatment to the time of progression, death, or the last follow-up. Differences in survival were tested using the log-rank test and represented by the Kaplan–Meier estimator plot. A 2-tailed *p* value ≤ 0.05 was considered to be statistically significant. Statistical analysis was performed using Sigma Plot version 14.0 (Systat Software, San Jose, CA, USA) and GraphPad version 8.0 for Windows (GraphPad Software Inc., San Diego, CA, USA). A ROC curve was calculated to determine the best cut-off value to dichotomize mutant ctDNA concentration to predict response to treatment. Multiple cut-offs were used, including those previously tested^25,27,36,66^ and those calculated by averaging two consecutive ctDNA values. The suitability of the cut-off was confirmed with Fisher’s exact test applied to the categories of responders and non-responders. The threshold for statistical significance was set at *p* ≤ 0.05.

### Reporting summary

Further information on research design is available in the Nature Research Reporting Summary linked to this article.

### Supplementary information

supplementary material

REPORTING SUMMARY

## Data Availability

The data are available from the corresponding authors on reasonable request. NGS raw data analyzed in this study have been deposited in the NCBI Sequence Read Archive database under BioProject ID PRJNA1085531 and are available at the following link, https://www.ncbi.nlm.nih.gov/sra/PRJNA1085531.
