# Supplementary material for: A multiparameter liquid biopsy approach allows to track melanoma dynamics and identify early treatment resistance
Source: NPJ Precis Oncol. 2024 Mar 28;8:78. doi: 10.1038/s41698-024-00567-0 (PMC10978909; doi:10.1038/s41698-024-00567-0)
Supplement: Supplementary file 1 — supplementary material [file 41698_2024_567_MOESM1_ESM.pdf]

Supplementary Material – Supplementary Figures

Supplementary Figure 1

Time point / Patient ID	T0	T1	T2	P
1	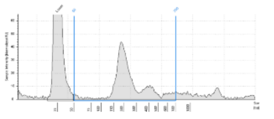	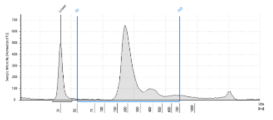	NA	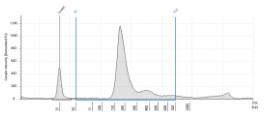
3	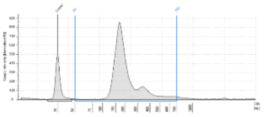	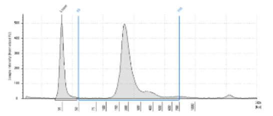	ND	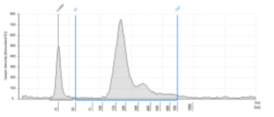
6	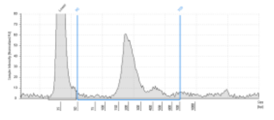	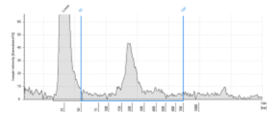	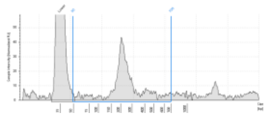	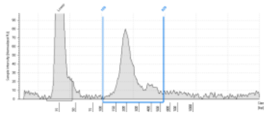
8	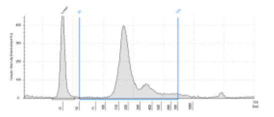	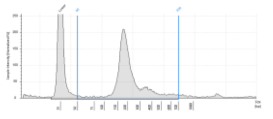	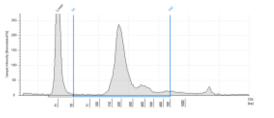	ND
9	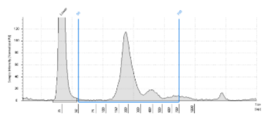	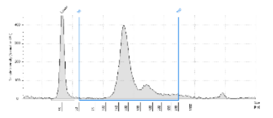	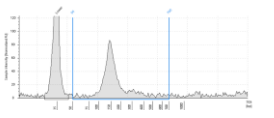	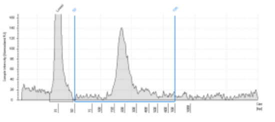
10	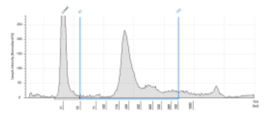	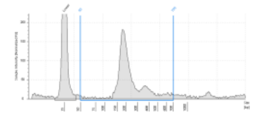	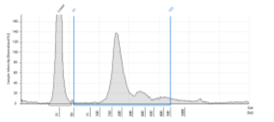	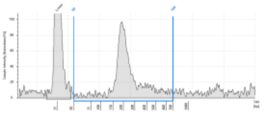
12	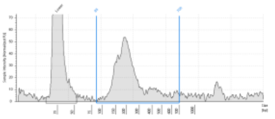	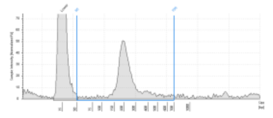	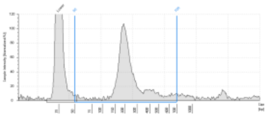	NA
13	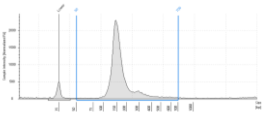	NA	NA	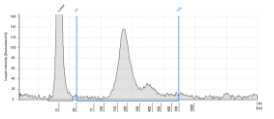
15	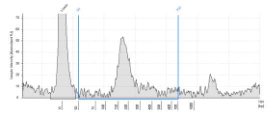	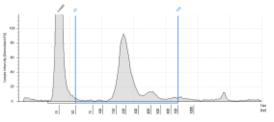	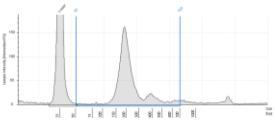	NA
17	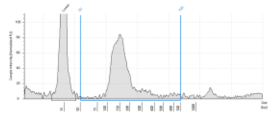	NA	NA	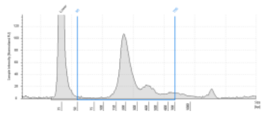
19	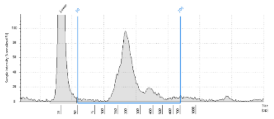	NA	NA	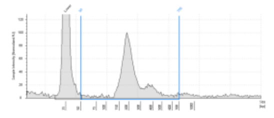

**24**	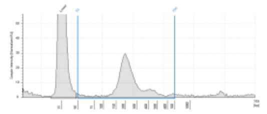	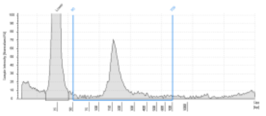	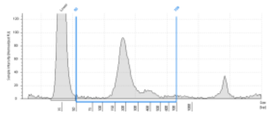	NA
**27**	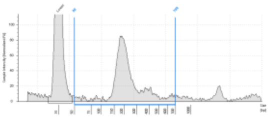	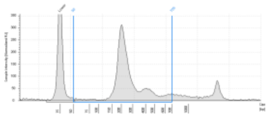	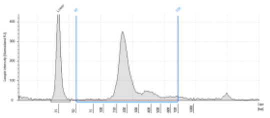	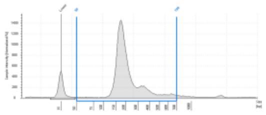
**28**	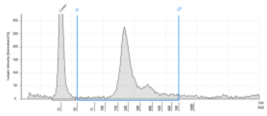	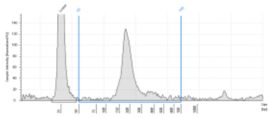	NA	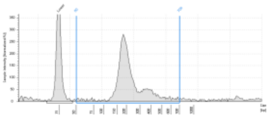
**34**	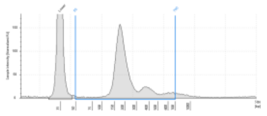	NA	NA	ND
**48**	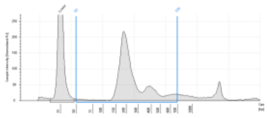	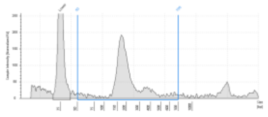	NA	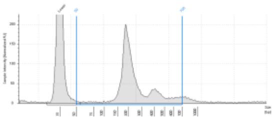
**49**	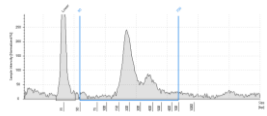	NA	NA	ND

TapeStation 4200 profiles of cfDNA. Electropherograms displaying a typical shape of a cfDNA trace, with most specimens showing one nucleosome peak, and a minority having a smear in the region of the tri-nucleosome peak. The presence of high molecular weight DNA is minimal for all samples.

Supplementary Figure 2

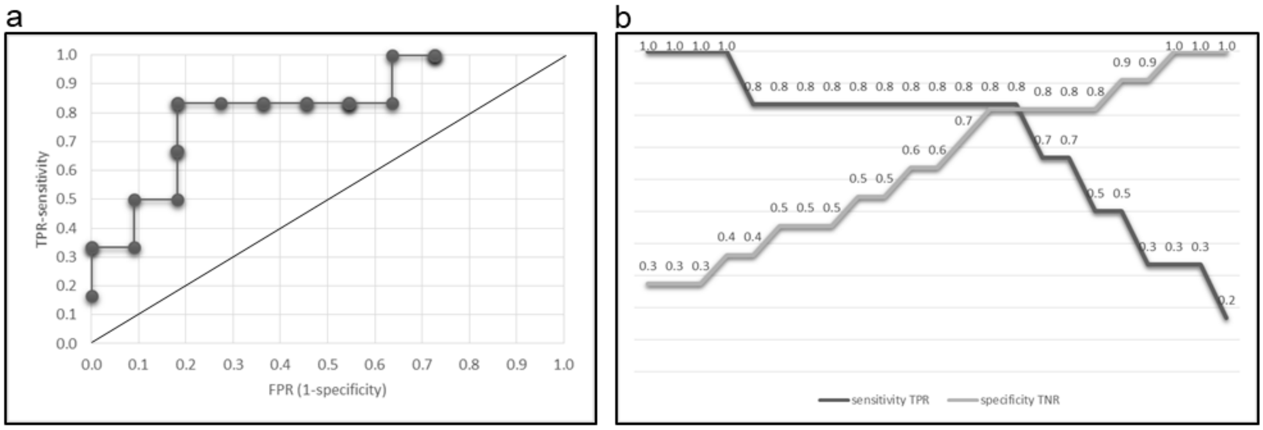

Cut-off	false positive rate	sensitivity	specificity
	FPR	TPR	TNR
0	0.7	1.0	0.3
0.85	0.7	1.0	0.3
1.5	0.7	1.0	0.3
2	0.6	1.0	0.4
5	0.6	0.8	0.4
10	0.5	0.8	0.5
12.7	0.5	0.8	0.5
15	0.5	0.8	0.5
20	0.5	0.8	0.5
59	0.5	0.8	0.5
100	0.4	0.8	0.6
114	0.4	0.8	0.6
150	0.3	0.8	0.7
216	0.2	0.8	0.8
283	0.2	0.8	0.8
500	0.2	0.7	0.8
550	0.2	0.7	0.8
1000	0.2	0.5	0.8
1931	0.1	0.5	0.9
3692	0.1	0.3	0.9
7571	0.0	0.3	1.0
10000	0.0	0.3	1.0
12100	0.0	0.2	1.0

BRAF-mutant ctDNA cut-off identification for discriminating responders from non-responders/early progressing patients. a) ROC curve. b) line-plot for cut-off identification. FPR, false positive rate; TPR, true positive rate; TNR, true negative rate.

Supplementary Figure 3

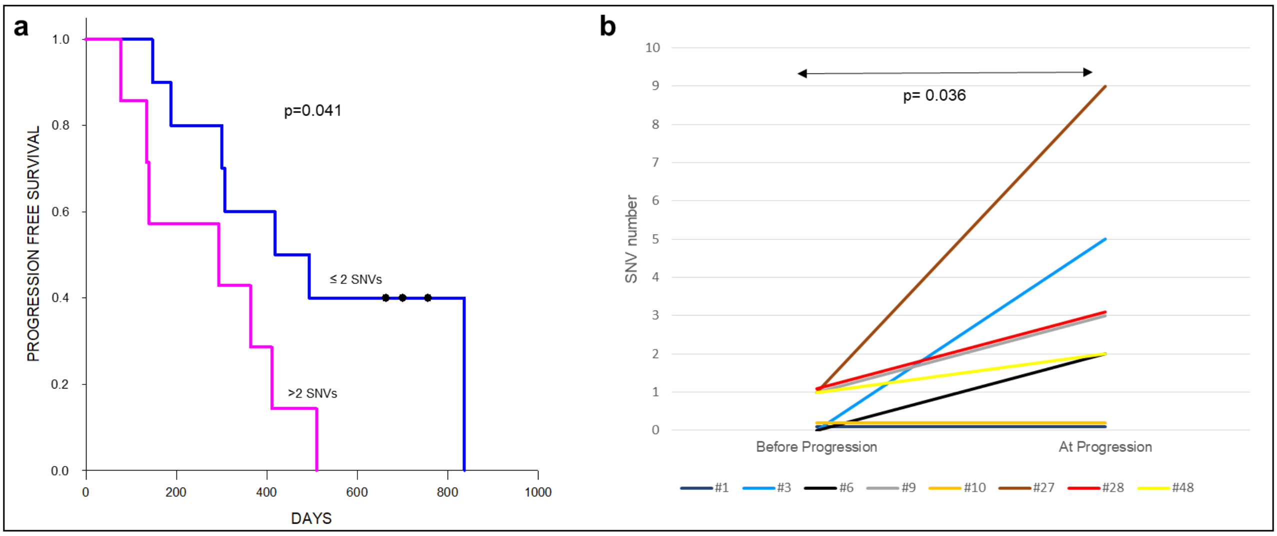

cfDNA Single Nucleotide Variant (SNV) load reflects tumor burden. (a) Significant correlation between baseline ctDNA SNV load and PFS (Log-Rank test, p=0.041); (b) Significant difference in SNV number before and at disease progression (matched sample t-test, p=0.036).

Supplementary Figure 4

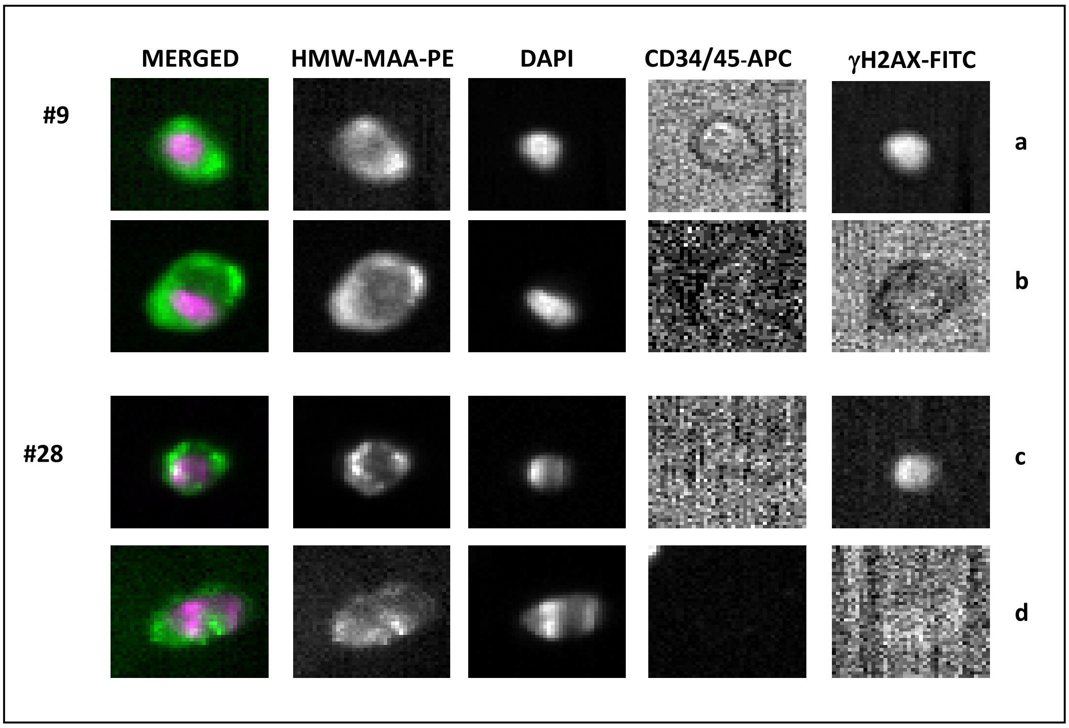

Representative images of γH2AX-positive (panels a and c) and γH2AX-negative (panels b and d) CMCs from patient #9 (panels a-b) and patient #28 (panels c-d) enriched and detected by the CellSearch system. Fluorophore-conjugated antibodies directed to High Molecular Weight Melanoma Associated Antigen (HMW-MAA-PE), CD34-APC and CD45-APC for endothelial cells and leukocytes, respectively, and γH2AX-FITC for DNA-damaged cells, were used. DAPI was used to stain nuclei. No staining was observed in the APC channel in the presence of a CMC, and the γH2AX-signal was considered as specific only when colocalizing with DAPI staining.

Supplementary Figure 5

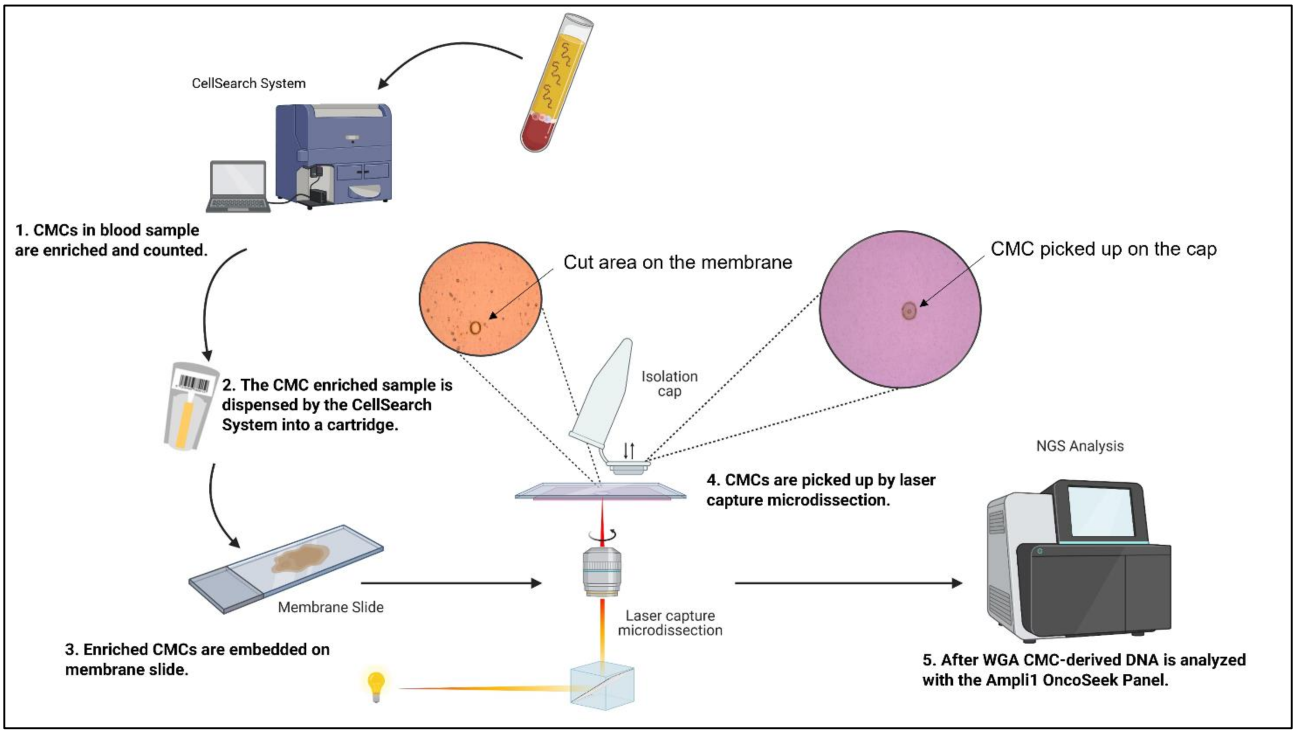

Workflow for CMC enrichment, microdissection, capture process, and genetic analysis. The workflow was created with BioRender (https://biorender.com/).

Supplementary Figure 6

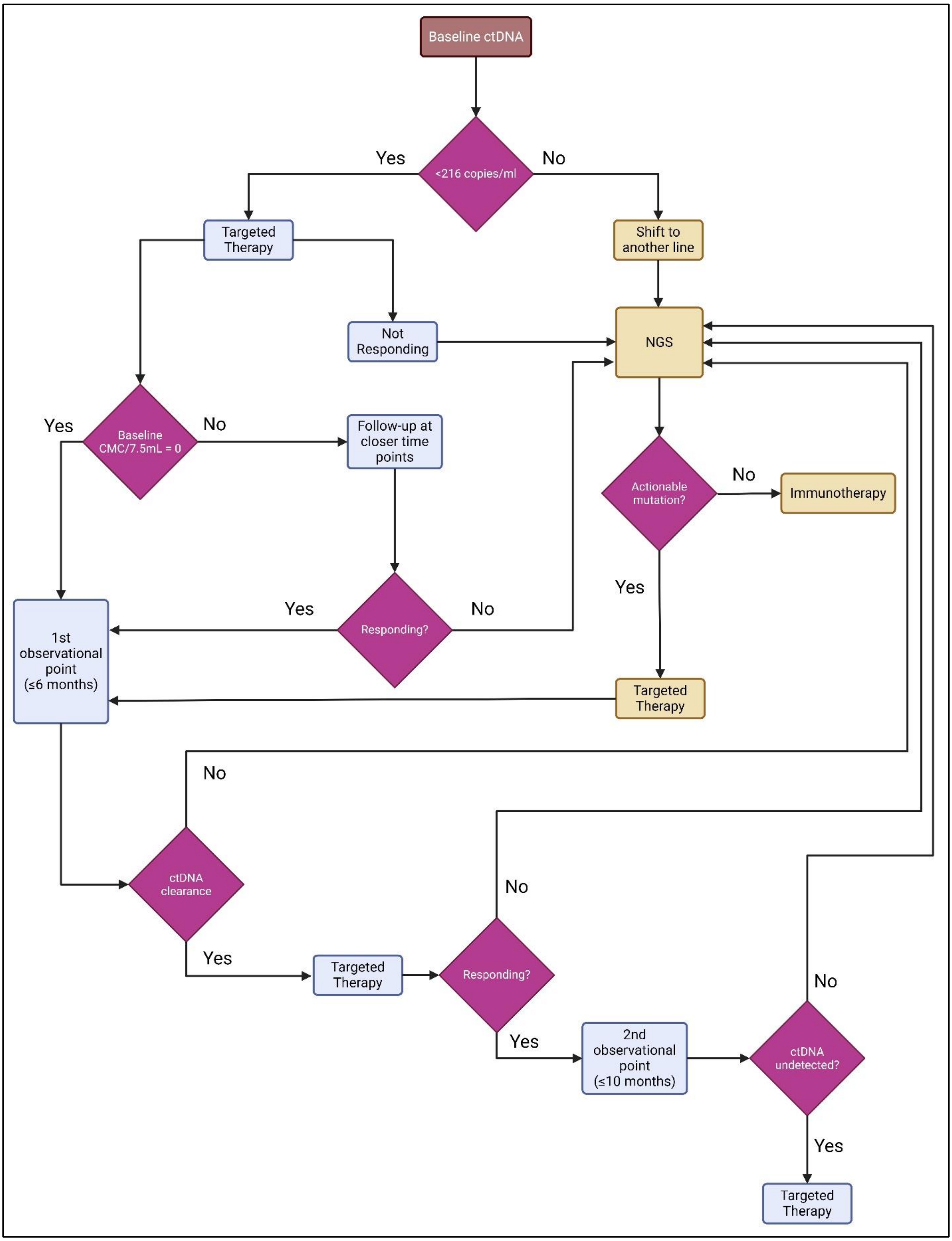

Temptative flowchart for the management of BRAF-mutant stage IV melanoma patients. The ctDNA cut-off value is proposed to shift patients, prior to the initiation of targeted therapy, toward an alternative line that is guided by NGS analysis. The number of CMCs at baseline helps in defining the closeness between observational follow-up points, and ctDNA clearance guides toward maintenance of targeted therapy or change in the therapy line. The flow chart was created with BioRender (https://biorender.com/).

Supplementary Material – Supplementary Tables

Supplementary Table 1

Time point / Patient ID	T0 (BRAF MAF%)	T1 (BRAF MAF%)	T2 (BRAF MAF%)	P (BRAF MAF%)	T0 (pTERT MAF%)	T1 (pTERT MAF%)	T2 (pTERT MAF%)	P (pTERT MAF%)
**1**	0.44 (V600E)	UD	NA	0.02	UD	ND	NA	UD
**3**	72.14 (V600K)	0.21	ND	50.38	UD	UD	ND	UD
**6**	0.08 (V600E)	UD	UD	5.17	UD	UD	UD	0.31 (C228T)
**8**	17.37 (V600E)	UD	UD	ND	1.57 (C228T)	ND	UD	ND
**9**	7.12 (V600E)	UD	0.26	3.78	2.51 (C228T)	UD	0.43	1.18
**10**	UD	UD	UD	UD	UD	UD	UD	UD
**12**	10.08 (V600E)	UD	UD	NA	2.37 (C250T)	ND	UD	NA
**13**	21.20 (V600E)	NA	NA	72.53	18.07 (C228T)	NA	NA	18.57
**15**	UD	UD	UD	NA	UD	ND	UD	NA
**17**	15.20 (V600E)	NA	NA	UD	6.00 (C250T)	NA	NA	UD
**19**	16.46 (V600E)	NA	NA	43.65	4.92 (C228T)	NA	NA	9.34
**24**	UD	UD	UD	NA	UD	ND	UD	NA
**27**	6.69 (V600K)	12.05	UD	74.53	0.87 (C228T)	2.16	UD	4.89
**28**	43.81 (V600E)	UD	NA	97.98	12.06 (C228T)	UD	NA	8.10
**34**	0.06 (V600E)	NA	NA	ND	0.08 (C250T)	NA	NA	ND
**48**	0.72 (T599dup)	UD	ND	0.28	0.26 (C250T)	UD	UD	0.18
**49**	36.74 (V600E)	ND	ND	ND	UD	ND	ND	ND

$BRAF^{V600E/K}$ and $pTERT^{C228T/C250T}$ mutant allele fraction (MAF) detected by ddPCR in serial plasma samples from 17 melanoma patients. Abbreviation: UD, undetectable; NA, not appropriate; ND, not done; T0, baseline; T1, 6 months follow-up; T2, 10 months follow-up; P, progression.

Supplementary Table 2

Patient ID	Time point	Total (ng)	On target	Median depth	Duplicates	1000X targets	500X bases
1	T1	40.3	71.2	2567.0	57.7	97.4	97.6
	P	41.4	70.7	2630.0	57.7	97.3	97.6
3	T0	50.0	77.7	3500.0	53.1	97.4	97.6
	T1	27.0	67.3	2321.0	61.4	97.3	97.6
	P	61.6	77.9	3860.0	52.7	97.5	97.7
8	T0	51.5	69.9	2880.0	59.6	97.6	97.7
	T1	27.6	62.6	2050.0	64.4	97.1	97.5
	T2	26.3	68.8	2235.0	62.0	97.3	97.4
10	T0	28.2	63.7	2084.0	59.9	97.5	97.6
13	T0	45.0	74.7	3027.0	48.4	97.5	97.7
	T2[£]	38.4	76.5	3197.0	53.3	97.5	97.7
	T3[£]	52.2	80.6	2041.0	34.7	96.3	96.5
15	T1	26.1	46.4	1325.0	61.8	96.3	97.0
27	T1	27.8	65.4	2192.0	58.3	97.4	97.6
	T2	28.3	60.3	1779.0	61.5	97.0	97.5
	P	47.6	75.5	3261.0	54.8	97.5	97.7
28	T0	27.0	61.8	2289.0	63.2	97.4	97.6
	P	26.6	72.1	2243.0	63.5	97.2	97.5
49	T0	28.5	56.6	1614.0	64.3	96.7	97.2
**Median (25-62 ng)**		**28.5**	**69.9**	**2289.0**	**59.6**	**97.4**	**97.6**
1	T0	17.7	43.0	734.0	76.8	73.8	90.1
6	T0	19.3	44.1	920.0	73.0	92.8	94.6
	P	18.0	49.5	970.0	79.7	94.3	95.7
9	T0	18.4	47.8	974.0	72.6	94.5	95.9
	T1	10.3	51.9	1087.0	72.9	94.6	95.9
	T2	17.6	49.1	1298.0	70.9	96.5	97.0
	P	15.0	56.7	1817.0	65.9	97.2	97.5
10	T1	21.0	49.3	1637.0	54.3	96.7	97.3
	T2	12.3	50.7	1151.0	71.7	95.3	96.3
	P	16.5	49.7	1493.0	67.7	97.1	97.4
12	T1	14.6	40.8	713.0	77.2	68.1	87.2
	T2	18.6	60.7	1301.0	71.5	95.3	96.7
13	P	13.1	63.9	1744.0	58.2	96.7	97.1
15	T0	21.1	37.5	979.0	78.2	95.3	96.3
	T2	15.7	54.0	1351.0	64.8	96.4	97.0
17	T0	10.8	57.3	1099.0	72.1	93.3	95.3
	P	11.5	53.4	1048.0	74.6	94.0	95.7
19	T0	11.4	52.8	1213.0	72.0	94.4	96.2
	P	12.0	58.3	1406.0	70.7	95.8	96.8
24	T0	12.7	36.9	435.0	71.9	16.5	23.3
	T1	11.4	37.9	805.0	75.1	82.0	90.5
	T2	14.8	32.3	819.0	70.3	85.8	92.0
27	T0	21.3	50.9	1398.0	68.5	95.5	96.8
28	T1	24.2	49.0	1178.0	70.1	95.1	96.4
34	T0	10.8	53.6	1094.0	74.0	94.8	96.1
48	T0	24.5	48.9	1149.0	70.7	94.8	96.3
	T1	23.2	58.0	1726.0	63.1	97.2	97.5
	P	15.5	54.3	1772.0	66.4	97.3	97.6
**Median (10-24 ng)**		**15.6**	**50.2**	**1150**	**71.6**	**94.9**	**96.3**
6	T1	6.4	35.0	446.0	79.9	17.9	26.4
	T2	7.2	34.9	464.0	82.6	20.8	34.9
12	T0	7.0	16.5	616.0	49.3	47.2	75.2
**Median (6-9 ng)**		**7.0**	**34.9**	**464.0**	**79.9**	**20.8**	**34.9**

Sequencing QC metrics from the SureSelect All In One Custom panel. 50 samples, belonging to the melanoma patient cohort, were used to test the customized sequencing workflow. Samples were stratified based on the initial amount of cfDNA: 25-62 ng, 10-24 ng or 6-9 ng. Abbreviations: 1000X targets, target regions covered at least at 1000X; 500X bases, bases of target regions covered at least at 500X; T0, baseline; T1, 6 month follow-up; T2, 10 month follow-up; P, progression; [£]additional samples corresponding to therapy change after progression (T2), and one month later (T3)[1].

Supplementary Table 3

Table 3.1

Sample ID	Gene	Variant	Expected MAF%	Detected MAF %		Mean	Var.	St. Dev.	St. Err.
				Replicate 1	Replicate 2				
HORIZON 5%	EGFR	L858R	5.0	4.40	5.40	4.90	0.500	0.707	0.500
	EGFR	ΔE746-A750	5.0	2.60	3.60	3.10	0.500	0.707	0.500
	EGFR	T790M	5.0	4.20	4.20	4.20	0.000	0.000	0.000
	EGFR	V769-D770insASV	5.0	3.10	3.40	3.25	0.045	0.212	0.150
	KRAS	G12D	6.3	5.40	4.80	5.10	0.180	0.424	0.300
	NRAS	Q61K	6.3	7.00	6.70	6.85	0.045	0.212	0.150
	NRAS	A59T	6.3	6.60	7.10	6.85	0.125	0.354	0.250
	PIK3CA	E545K	6.3	7.00	6.10	6.55	0.405	0.636	0.450

Table 3.2

Sample ID	Gene	Variant	Expected MAF %	Detected MAF %			Mean	Var.	St. Dev.	St. Err.
				Replicate 1	Replicate 2	Replicate 3				
HORIZON 1%	EGFR	L858R	1.0	0.92	1.10	1.20	1.07	0.020	0.142	0.082
	EGFR	ΔE746-A750	1.0	UD	0.41	0.36	0.39	0.001	0.035	0.020
	EGFR	T790M	1.0	0.72	0.89	0.85	0.82	0.008	0.089	0.051
	EGFR	V769-D770insASV	1.0	0.45	0.75	0.41	0.54	0.035	0.186	0.107
	KRAS	G12D	1.3	0.90	1.10	1.50	1.17	0.093	0.306	0.176
	NRAS	Q61K	1.3	0.91	1.30	1.10	1.10	0.038	0.195	0.113
	NRAS	A59T	1.3	1.30	1.20	0.82	1.11	0.064	0.253	0.146
	PIK3CA	E545K	1.3	1.20	1.20	1.20	1.20	0.000	0.000	0.000

Table 3.3

Sample ID	Gene	Variant	Expected MAF %	Detected MAF %
HORIZON WT	EGFR	L858R	0.0	0.0
	EGFR	ΔE746-A750	0.0	0.0
	EGFR	T790M	0.0	0.0
	EGFR	V769-D770insASV	0.0	0.0
	KRAS	G12D	0.0	0.0
	NRAS	Q61K	0.0	0.0
	NRAS	A59T	0.0	0.0
	PIK3CA	E545K	0.0	0.0

Performance of the SureSelect All in One custom panel in the correct detection of several mutations. A certified control with mutations at 5/6.3% (Table 3.1) and 1.3/1% (Table 3.2) MAFs and a WT certified control (Table 3.3) were run in two and three replicates, respectively. Abbreviations: UD, undetectable.

Supplementary Table 4

Patient ID	cfDNA time point	Total (ng)	ddPCR mutation detection	SureCall mutation detection	Filtered Read Depth	Coverage
**1**	T0	17.7	BRAF V600E 0.44%	BRAF V600E 1.00%	739	8.13
	P	41.4	BRAF V600E 0.03%	BRAF V600E 0.10%*	NA	NA
**3**	T0	50.0	BRAF V600K 72.10%	BRAF V600K 67.60%	3847	2600.57
			FBXW7 R465C 16.60%	FBXW7 R465C 16.30%	3441	560.88
	T1	27.0	BRAF V600K 0.21%	BRAF V600K 0.30%*	NA	NA
	P	61.6	BRAF V600K 50.38%	BRAF V600K 44.90%	4335	1946.42
			FBXW7 R465C 10.40%	FBXW7 R465C 9.70%	3953	383.44
**5**	P	19.9	GNAQ Q209L 2.50%	GNAQ Q209L 1.10%	913	10.04
**6**	T0	19.3	BRAF V600E 0.08%	UD	NA	NA
	P	18.4	BRAF V600E 5.17%	BRAF V600E 6.60%	1113	73.46
**8**	T0	51.5	BRAF V600E 17.40%	BRAF V600E 17.10%	3045	520.70
**9**	T0	18.4	BRAF V600E 7.12%	BRAF V600E 8.30%	988	82.00
	T2	17.6	BRAF V600E 0.30%	BRAF V600E 0.50%*	NA	NA
	P	15.0	BRAF V600E 3.78%	BRAF V600E 4.60%	1658	76.27
			NRAS Q61L 2.70%	NRAS Q61L 3.60%	1680	60.48
**11**	T0	24.7	GNA11 Q209L 0.22%	UD	NA	NA
	P	22.8	GNA11 Q209L 3.30%	GNA11 Q209L 3.40%	2148	73.03
**12**	T0	7.0	BRAF V600E 10.08%	BRAF V600E 18.20%	652	118.66
**13**	T0	45.0	BRAF V600E 21.20%	BRAF V600E 20.60%	3308	681.45
			MEK1 P124L 20.40%	MEK1 P124L 23.70%	3205	759.59
	P	13.1	BRAF V600E 72.50%	BRAF V600E 73.10%	4800	3508.80
			MEK1 P124L 28.44%	MEK1 P124L 27.70%	1883	521.59
**17**	T0	10.8	BRAF V600E 15.20%	BRAF V600E 25.70%	1362	350.03
**19**	T0	11.4	BRAF V600E 16.46%	BRAF V600E 24.70%	1358	335.43
			MEK1 P124L 13.78%	MEK1 P124L 17.60%	1365	240.24
	P	12.0	BRAF V600E 43.65%	BRAF V660E 42.50%	1860	790.50
			MEK1 P124L 22.99%	MEK1 P124L 21.50%	1579	339.49
			GNA11 Q209L 0.8%	GNA11 Q209L 1.20%	1389	16.67
**27**	T0	21.3	BRAF V600K 6.69%	BRAF V600K 5.00%	1368	68.40
			RAC1 P29S 3.0%	RAC1 P29S 2.40%	836	20.06
			RET S705F 5.0%	RET S705F 3.70%	1512	55.94
	T1	27.8	BRAF V600K 12.05%	BRAF V600K 11.20%	2261	253.23
			RAC1 P29S 6.70%	RAC1 P29S 6.50%	1620	105.30
			TP53 R175H 2.50%	TP53 R175H 2.10%	2473	51.93
			RET S705F 2.60%	RET S705F 3.40%	1713	58.24
	T2	28.3	TP53 R175H 3.50%	TP53 R175H 3.30%	1896	62.57
	P	47.6	BRAF V600K 74.53%	BRAF V600K 71.50%	7323	5235.95
			KRAS G12A 11.99%	KRAS G12A 9.50%	2772	263.34
			RAC1 P29S 24.4%	RAC1 P29S 18.30%	2747	502.70
			TP53 R175H 2.10%	TP53 R175H 1.80%	3025	54.45
			RET S705F 13.60%	RET S705F 13.10%	1841	241.17
**28**	T0	27.0	BRAF V600E 43.81%	BRAF V600E 40.20%	2905	1167.81
			PIK3CA E542K 0.73%	PIK3CA E542K 0.98%	2239	21.94
			PIK3CA E545K 7.40%	PIK3CA E545K 6.70%	2254	151.02
	P	26.6	BRAF V600E 97.98%	BRAF V600E 97.70%	23655	23110.94
			PIK3CA E542K 13.70%	PIK3CA E542K 12.50%	2387	298.38
**29**	T0	31.1	BRAF V600E 12.28%	BRAF V600E 11.90%	2469	293.81
**34**	T0	10.8	BRAF V600E 0.06%	UD	NA	NA
**48**	T0	24.5	BRAF T599dup 0.72%	BRAF T599dup 0.64%*	NA	NA
	P	15.5	BRAF T599dup 0.28%	BRAF T599dup 0.33%*	NA	NA
**49**	T0	28.5	BRAF V600E 36.70%	BRAF V600E 41.90%	2157	903.78

Testing and validation of the ability of the SureSelect All In One panel to correctly detect the MAF of several SNVs by means of ddPCR as an orthogonal technique. The validation test has been performed on 31 samples, 28 belonging to the melanoma cohort and 3 extra samples. Total (ng): initial amount of cfDNA; Filtered Read Depth: read depth at the mutation site; Coverage: number of reads supporting the mutant allele. Abbreviations: UD, undetectable; NA, not available; T0, baseline; T1, 6 months follow-up; T2, 10 months follow-up; P, progression; * call performed from the inspection of the bam file.

Supplementary Table 5

Time point	cfDNA MAF % - NGS	cfDNA MAF % - ddPCR	blood cell DNA MAF % - ddPCR
**T0**	1.80	ND	1.83
**T1**	2.10	2.47	2.69
**T2**	3.30	3.53	3.04
**P**	1.80	2.03	3.03

TP53 p.R175H allele frequency. MAF was detected by NGS (column 2) and by ddPCR in cfDNA (column 3) and matched DNA extracted from blood cells (column 4) of patient #27. Abbreviations: ND, not done; T0, baseline; T1, 6 months follow-up; T2, 10 months follow-up; P, progression.

Supplementary Table 6

Patient ID	cfDNA time points	Partial/ Complete CNV detection	Position (cytoband 7q34)	Type	Size	Surecall CN assessment	CN assessment (n. of copies)	ddPCR CN assessment		Final CN assessment	Concordance (Y/N)
								BRAF/ CHR14	BRAF/ CHR7		
1	T0	Partial	7:140.734.648-140.886.119	Gain	151.5 Kb	3	Diploid	2.1	/	Diploid	Y
	T1	No variation detected	NA	NA	NA	2	Diploid	2.1	/	Diploid	Y
	P	No variation detected	NA	NA	NA	2	Diploid	1.9	/	Diploid	Y
3	T0	Complete	7:140.539.751-141.072.685	Gain	539.9 Kb	3	Gain (3)	2.7	1.9	Gain/Polysomy	Y
	T1	No variation detected	NA	NA	NA	2	Diploid	2.0	2.1	Diploid	Y
	P	No variation detected	NA	NA	NA	2	Diploid	2.1	1.9	Diploid	Y
6	T0	Partial	7:140.570.473-140.886.119;	Gain	315.6 Kb	3	Diploid	2.2	2.2	Diploid	Y
	T1	Partial	7:140.734.648-140.860.257	Gain	125.6 Kb	3	Diploid	1.9	/	Diploid	Y
	T2	Partial	7:140.739.901-140.924.711	Gain	98.5 Kb	3	Diploid	1.7	2.5	Diploid	Y
	P	Complete	7:140.700.818-140.924.711	Gain	223.9 Kb	3	Gain (3)	2.7	2.0	Gain/Polysomy	Y
8	T0	No variation detected	NA	NA	NA	2	Diploid	2.4	/	Diploid	Y
	T1	No variation detected	NA	NA	NA	2	Diploid	2.3	/	Diploid	Y
	T2	No variation detected	NA	NA	NA	2	Diploid	2.1	/	Diploid	Y
9	T0	Partial	7:140.700.818-140.886.119	Gain	185.3 Kb	3	Diploid	1.8	/	Diploid	Y
	T1	No variation detected	NA	NA	NA	2	Diploid	2.1	/	Diploid	Y
	T2	Partial	7:140.726.418-140.924.711	Gain	198.3 Kb	3	Diploid	2.2	/	Diploid	Y
	P	No variation detected	NA	NA	NA	2	Diploid	2.0	2.2	Diploid	Y
10	T0	No variation detected	NA	NA	NA	2	Diploid	2.2	/	Diploid	Y
	T1	Partial	7:140.735.225-140.924.711	Gain	189.5 Kb	3	Diploid	2.0	/	Diploid	Y
	T2	Partial	7:140.735.225-140.886.119	Gain	150.9 Kb	3	Diploid	2.0	/	Diploid	Y
	P	No variation detected	NA	NA	NA	2	Diploid	2.0	2.0	Diploid	Y
12	T0	Partial	7:140.734.648-140.886.119	Gain	151.5 Kb	3	Diploid	2.4	/	Diploid	Y
	T1	Partial	7:140.734.648-140.886.119	Gain	151.5 Kb	3	Diploid	2.0	/	Diploid	Y
	T2	Partial	7:140.570.473-140.886.119;	Gain	315.6 Kb	3	Diploid	2.3	/	Diploid	Y
13	T0	No variation detected	NA	NA	NA	2	Diploid	2.0	1.9	Diploid	Y
	P	Complete	7:140.691.748-140.924.711	Gain	233.0 Kb	8	Amp (8)	6.5	5.7	Amp/disomy	Y
15	T0	Partial	7:140.749.946-140.860.257	Gain	110.3 Kb	3	Diploid	2.3	/	Diploid	Y
	T1	Partial	7:140.734.648-140.886.119	Gain	151.5 Kb	3	Diploid	2.2	/	Diploid	Y
	T2	Partial	7:140.756.204-140.886.119	Gain	129.9 Kb	3	Diploid	2.0	/	Diploid	Y
17	T0	Complete	7:140.539.751-140.886.119; 7:140.539.751-141.118.682;	Gain	578.9 Kb	3	Gain (3)	2.7	1.6	Gain/polysomy	Y
	P	Partial	7:140.679.385-140.749.503	Gain	70.1 Kb	3	Diploid	1.7	1.8	Diploid	Y
19	T0	Partial	7:140.539.751-140.886.119	Gain	346.4 Kb	3	Diploid	2.5	2.3	Diploid	Y
	P	Complete	7:140.539.751-141.118.682	Gain	578.9 Kb	3	Gain (3)	2.9	2.9	Gain/disomy	Y
24	T0	Partial	7:140.756.204-140.924.711	Gain	168.5 Kb	3	Diploid	2.3	/	Diploid	Y
	T1	Partial	7:140.739.901-140.886.119	Gain	146.2 Kb	3	Diploid	2.3	2.1	Diploid	Y
	T2	Partial	7:140.734.648-140.886.119	Gain	151.5 Kb	3	Diploid	2.3	/	Diploid	Y
27	T0	Partial	7:140.570.473-140.740.031; 7:140.808.959-140.886.119	Gain	169.6 Kb; 77.2 Kb	3	Diploid	2.3	/	Diploid	Y
	T1	Partial	7:140.734.648-140.886.119	Gain	151.5 Kb	3	Diploid	2.2	/	Diploid	Y
	T2	No variation detected	NA	NA	NA	2	Diploid	2.1	/	Diploid	Y
	P	Complete	7:140.539.751-141.118.682	Gain	578.9 Kb	7	Amp (7)	6.5	5.6	Amp/disomy	Y
28	T0	Complete	7:140.539.751-140.886.119;	Gain	346.4 Kb;	3	Gain (3)	3.1	1.9	Gain/polysomy	Y
	T1	Partial	7:140.734.648-140.834.760	Gain	100.1 Kb	3	Diploid	2.1	/	Diploid	Y
	P	Complete	7:140.539.751-141.118.682	Gain	578.9 Kb	24	Amp (24)	81.3	49.0	Amp/polysomy	Y
34	T0	No variation detected	NA	NA	NA	2	Diploid	2.4	/	Diploid	Y

Patient ID	cfDNA time points	Partial/ Complete CNV detection	Position (cytoband 7q34)	Type	Size	Surecall CN assessment	CN assessment (n. of copies)	ddPCR CN assessment		Final CN assessment	Concordance (Y/N)
								BRAF/ CHR14	BRAF/ CHR7		
48	**T0**	Partial	7:140.570.473-140.886.119	Gain	315.6 Kb	3	Diploid	2.1	/	Diploid	Y
	**T1**	No variation detected	NA	NA	NA	2	Diploid	2.1	/	Diploid	Y
	**P**	Partial	7:140.558.055-140.886.119	Gain	328.1 Kb	3	Diploid	2.3	1.8	Diploid	Y
49	**T0**	Complete	7:140.539.751-140.924.711	Gain	385.0 Kb	3	Gain (3)	2.3	2.2	Diploid	N

Validation of copy number variation (CNV) assessment by SureSelect All In One custom panel. The CNV detected by the panel (columns 3-8) was orthogonally validated by ddPCR, which was performed with 2 different references (columns 9-11). The final copy number assessment and output concordance are reported in columns 12 and 13, respectively. Partial/complete CNV detection: partial or complete agreement of probes covering the BRAF region in calling a copy number variation. The ddPCR cut-off for calling a copy number alteration was 2.16 ± 0.39 for BRAF/CHR14 and 2.03 ± 0.56 for BRAF/CHR7. Abbreviations: CN, copy number; NA, not applicable; Amp, amplification; Y/N, yes/no; T0, baseline; T1, 6 months follow-up; T2, 10 months follow-up; P, progression.

Supplementary Table 7

Patient ID	Gene	Chromo-somal location	Position (hg38)	nt change	AA change	Type of change	COSMIC ID	cfDNA time points (MAF %)			Present in tumor δ
								T0	T1	P	
**1**	BRAF	7q34	140753336	T>A	V600E	Nonsynonymous	COSM476	1.1	-	-	Y
**6**	ABL1	9q34.12	130854948	G>A	R153H	Nonsynonymous	COSM6934804	1.1	-	-	Y
	BRAF	7q34	140753336	T>A	V600E	Nonsynonymous	COSM476	-	-	6.6	Y
	RET	10q11.21	43124939	C>T	A999V	Nonsynonymous	COSM6240510	1.1	-	-	N
	SF3B1	2q33.1	197418534	G>A	R157Q	Nonsynonymous	COSV59214718	1.3	-	-	N
	SF3B1	2q33.1	197409962	C>T	R238C	Nonsynonymous	COSM4506189	-	-	1.3	Y
**12**	ARID2	12q12	45850473	C>T	P784S	Nonsynonymous	COSM5413317	14.7	-	-	Y
	BRAF	7q34	140753336	T>A	V600E	Nonsynonymous	COSM476	18.2	-	-	Y
**19**	BRAF	7q34	140753336	T>A	V600E	Nonsynonymous	COSM476	24.7	NA	42.5	Y
	ERBB4	2q34	211383947	G>A	E1199K	Nonsynonymous	COSM4764537	11.1	NA	9.4	Y
	GNA11	19p13.3	3118944	A>T	Q209L	Nonsynonymous	COSM52969	-	NA	1.2	N
	GRIN2A	16p13.2	10180321	C>T	P31S	Nonsynonymous	COSM2141702	14.5	NA	21.8	Y
	KIT	4q12	54698386	C>T	S147F	Nonsynonymous	COSM5904789	15.7	NA	20.7	Y
	MEK1	15q22.31	66436825	C>T	P124L	Nonsynonymous	COSM1315861	17.6	NA	21.5	Y
	ROS1	6q22.1	117385697	G>A	G764R	Nonsynonymous	COSM3157846	16.7	NA	16.4	Y
**48**	ERBB4	2q34	211702093	G>A	A455T	Nonsynonymous	COSM7344043	5.1	2.2	2.3	Y
	PPP6C	9q33.3	125158280	C>T	H151Y	Nonsynonymous	COSM23144	-	-	0.8	Y
	RET	10q11.21	43124939	C>T	A999V	Nonsynonymous	COSM6240510	1.1	-	-	N
**49**	BRAF	7q34	140753336	T>A	V600E	Nonsynonymous	COSM476	41.9	NA	NA	Y
	MET	7q31	116769777	G>A	E906K	Nonsynonymous	COSM5576816	20.0	NA	NA	Y
	TP53	17p13.1	7674241	C>T	S241F	Nonsynonymous	COSM10812	44.5	NA	NA	Y

Parallel identification of cfDNA SNVs in tumor tissue. Abbreviations: AA, amino acid; cfDNA, cell-free DNA; COSMIC, Catalogue of Somatic Mutations in Cancer; N, no; Y, yes; nt, nucleotide; SNV, single nucleotide variant; MAF, mutant allele fraction; -, not detected; NA, not applicable. δ Matched tumor tissue was obtained at the surgery upon relapse or from the primary tumor.

Supplementary Table 8

#	Cell line	Cell line mutation	n. of cells	QC output	ddPCR output	Final assessment
F32	MDA-MB-231 (Orange)	KRAS G13D (MAF 50%)	1	Not passed	N/A	N/A
F33	MDA-MB-231 (Orange)	KRAS G13D (MAF 50%)	1	Passed	Mut (81%)	AI
F34	MDA-MB-231 (Orange)	KRAS G13D (MAF 50%)	1	Passed	Mut (87%)	AI
F35	MDA-MB-231(Orange)	KRAS G13D (MAF 50%)	1	Not passed	N/A	N/A
F36	MDA-MB-231(Orange)	KRAS G13D (MAF 50%)	1	Not passed	N/A	N/A
F37	MDA-MB-231(Orange)	KRAS G13D (MAF 50%)	1	Passed	Mut (50%)	OK
F38	MDA-MB-231(Orange)	KRAS G13D (MAF 50%)	1	Passed	Mut (100%)	AD
F39	MDA-MB-231(Orange)	KRAS G13D (MAF 50%)	1	Passed	Mut (37%)	AI
F40	MDA-MB-231(Orange)	KRAS G13D (MAF 50%)	1	Not passed	N/A	N/A
F41	MDA-MB-231(Orange)	KRAS G13D (MAF 50%)	1	Passed	NULL	N/A
F44	MDA-MB-231(Orange)	KRAS G13D (MAF 50%)	1	Passed	Mut (99%)	AI
F45	MDA-MB-231(Orange)	KRAS G13D (MAF 50%)	1	Passed	WT (100%)	AD
F46	MDA-MB-231(Orange)	KRAS G13D (MAF 50%)	1	Passed	WT (100%)	AD
F47	MDA-MB-231(Orange)	KRAS G13D (MAF 50%)	1	Passed	WT (100%)	AD
F48	MDA-MB-231(Orange)	KRAS G13D (MAF 50%)	1	Not passed	N/A	N/A
F59	Spike-in H1975 (kit CXC)	EGFR L858R (MAF 70%)	1	Not passed	N/A	N/A
F60	Spike-in H1975 (kit CXC)	EGFR L858R (MAF 70%)	2	Passed	Mut (50%)	AI
F61	Spike-in H1975 (kit CXC)	EGFR L858R (MAF 70%)	10	Passed	Mut (75%)	OK
F62	Spike-in H1975 (kit CXC)	EGFR L858R (MAF 70%)	5	Passed	Mut (47%)	AI
F63	Spike-in H1975 (kit CXC)	EGFR L858R (MAF 70%)	5	Passed	Mut (100%)	AD
F64	Spike-in H1975 (kit CXC)	EGFR L858R (MAF 70%)	2	Passed	Mut (99%)	AD
F65	Spike-in H1975 (kit CXC)	EGFR L858R (MAF 70%)	1	Passed	Mut (99%)	AD
F66	Spike-in H1975 (kit CXC)	EGFR L858R (MAF 70%)	1	Passed	Mut (65%)	OK
F67	Spike-in H1975 (kit CXC)	EGFR L858R (MAF 70%)	1	Passed	Mut (99.9%)	AD
F68	Spike-in H1975 (kit CXC)	EGFR L858R (MAF 70%)	10	Not passed	N/A	N/A
F69	Spike-in H1975 (kit CXC)	EGFR L858R (MAF 70%)	5	Passed	Mut (60%)	AI
F104	Spike-in H1975 (kit CTC)	EGFR L858R (MAF 70%)	10	Passed	Mut (65.3%)	OK
F105	Spike-in H1975 (kit CTC)	EGFR L858R (MAF 70%)	10	Passed	Mut (82%)	AI
F106	Spike-in H1975 (kit CTC)	EGFR L858R (MAF 70%)	1	Not passed	N/A	N/A
F107	Spike-in H1975 (kit CTC)	EGFR L858R (MAF 70%)	1	Passed	NULL	N/A
F108	Spike-in H1975 (kit CTC)	EGFR L858R (MAF 70%)	5	Passed	Mut (94%)	AI
F109	Spike-in H1975 (kit CTC)	EGFR L858R (MAF 70%)	5	Passed	Mut (69%)	OK
F111	Spike-in SK-MEL-23 (kit CMC)	BRAF G466A (MAF 13%)	1	Not passed	N/A	N/A
F112	Spike-in SK-MEL-23 (kit CMC)	BRAF G466A (MAF 13%)	1	Not passed	N/A	N/A
F113	Spike-in SK-MEL-23 (kit CMC)	BRAF G466A (MAF 13%)	6	Passed	Mut (10%)	OK
F117	MDA-MB-231 (Orange)	KRAS G13D (MAF 50%)	1	Passed	NULL	N/A
F118	MDA-MB-231 (Orange)	KRAS G13D (MAF 50%)	1	Passed	Mut (97%)	AI
F119	MDA-MB-231 (Orange)	KRAS G13D (MAF 50%)	1	Passed	WT (100%)	AD
F123	Spike-in H1975 (kit CTC)	EGFR L858R (MAF 70%)	1	Not passed	N/A	N/A
F124	Spike-in H1975 (kit CTC)	EGFR L858R (MAF 70%)	1	Not passed	N/A	N/A
F125	Spike-in H1975 (kit CTC)	EGFR L858R (MAF 70%)	1	Not passed	N/A	N/A
F185	Spike-in SK-MEL-28 (kit CMC)	BRAF V600E (MAF 100%)	5	Passed	Mut (100%)	N/A
F186	Spike-in di SKMEL28 (kit CMC)	BRAF V600E (MAF 100%)	11	Passed	Mut (100%)	N/A
F187	Spike-in di SKMEL28 (kit CMC)	BRAF V600E (MAF 100%)	1	Passed	Mut (97%)	N/A

Workflow validation from cell isolation to molecular analysis. Cells belonging to different cell lines were isolated, individually or in clusters subjected to WGA and subsequent QC and finally tested for the mutation of interest by ddPCR. **Column 1**: #, sample id; **column 2**: cell line name and treatment performed before microdissection; Orange: cells stained with the CellTracker Orange dye; kit CTC/CXC/CMC: type of CellSearch run performed before isolation; **column 3**: mutation carried by the cell line and its allelic fraction; **column 4**: number of cells isolated in each tube; **column 5**: results of QC test after WGA; **column 6**: outcome of ddPCR analysis; **column 7**: assessment of workflow performance after ddPCR analysis. Abbreviations: AD, allele dropout; AI, allelic imbalance; OK, allelic fraction correctly identified; N/A, not applicable; QC, quality control; MAF, mutant allele fraction; WT, wild type; Mut, mutant.

Supplementary Table 9

Gene	Hotspot mutation	SK-MEL-28	MCF7	H1975
BRAF	V600E	●		
CDK4	R24C			
EGFR	P753S	●		
EGFR	T790M			●
EGFR	L858R			●
PIK3CA	G118D			
PIK3CA	E545K		●	
PTEN	T167A			
TP53	L145R	●		
TP53	R273H			●
MYC AMPLIFICATION			●	●
EGFR AMPLIFICATION				●

● Hotspot mutations/CNA reported in cBioPortal and detected by Ampli1 OncoSeek Panel

Hotspot mutations/CNA reported in cBioPortal and not detected by Ampli1 OncoSeek Panel

Targeted sequencing with Ampli1 OncoSeek panel performed on SK-MEL-28, MCF7, and H1975 cancer cell lines. Expected and detected mutations, plus copy number alterations (CNA), are reported. SK-MEL-28 CNA analysis gave noisy results with unclear copy number assessment and was not included in the table.

Supplementary Table 10

Table 10.1

Gene	Chromo-somal location	Position (hg38)	nt change	AA change	Type of change	Variant annotation (COSMIC)	T0 ctDNA MAF (%)	T0 CMC MAF (%)	T2 ctDNA MAF (%)	T2 CMC MAF (%)
ADAMTS18	16q23.1	77364300	G>A	**G115E**	Nonsynonymous	COSM142238	34.8	NI	23.6	NI
BRAF	7q34	140753336-140753337	delinsAA	**V600K**	Nonsynonymous	COSM473	67.6*	99.8*	44.9*	-
FBXW7	4q31.3	152328233	C>T	**R465C**	Nonsynonymous	COSM22932	16.3	31.4	9.7	-
PREX2	8q13.2	68138502	G>A	**E1358K**	Nonsynonymous	COSM3650517	26.4	NI	18.3	NI
PTEN	10q23.31	87957958	T>G	**L247***	Stopgain	COSM3736942	52.0	99.1	25.1	-
TP53	17p13.1	7675227	G>A	**A129T**	Nonsynonymous	COSM44966	-	-	-	20.9

Table 10.2

Gene	Chromo-somal location	Position (hg38)	nt change	AA change	Type of change	Variant annotation (COSMIC)	T2 ctDNA MAF (%)	T2 CMC MAF (%)	T3 ctDNA MAF (%)	T3 CMC MAF (%)
ATM	11q22.3	108330374	C>T	**L2490F**	Nonsynonymous	COSM327924	27.6	OTR	7.8	OTR
BRAF	7q34	140753336	T>A	**V600E**	Nonsynonymous	COSM476	63.9*	23.6*	24.7*	-
ERBB4	2q34	211722479	T>C	**F266S**	Nonsynonymous	COSM6978929	-	100.0	-	-
MEK1	15q22.31	66436825	C>T	**P124L**	Nonsynonymous	COSM1315861	24.0*	ND*	9.1*	-
TP53	17p13.1	7675145	G>A	**R156H**	Nonsynonymous	COSM43739	-	17.9	-	NC

Targeted sequencing with Ampli1 OncoSeek panel performed on microdissected CMCs from patients #3 (Table 10.1) and #13 (Table 10.2). SNVs with relative MAFs detected from both ctDNA analysis and from microdissected CMCs are reported. Abbreviations: -, not detected; NC, not covered; NI, not included; OTR, out of target; *, SNV detected/confirmed by ddPCR.

Supplementary Material – Materials and Methods

cfDNA extraction and processing

The cfDNA was isolated from stored plasma using the QIAamp Circulating Nucleic Acid Kit (Qiagen, Hilden, Germany), and quantified on a Qubit Fluorometric Quantitation System 1.0 (Thermo Fisher Scientific, Waltham, MA, USA) following the manufacturer's instructions. ddPCR was performed in duplicate with 5-7 µl per well. NGS libraries were generated from 6-62 ng of cfDNA and sequenced on a NextSeq 550 system (Illumina, San Diego, CA, USA).

ddPCR analysis

Specific hotspots in BRAF gene and TERT promoter (pTERT) were assessed by ddPCR (BioRad, Hercules, CA, USA) together with SNVs detected by NGS (BioRad, Hercules, USA). The entire list of commercial and customized probes is reported in Table A. The reactions were performed in a 20 µl reaction mix containing 1× droplet PCR supermix, 250 nM of each probe, 450 nM of primers and 2-7 µL of cfDNA, which was quantified in parallel by ddPCR. Droplets were generated and analyzed using the QX200 system (BioRad). For both C228T and C250T pTERT assays, Na_{2}-EDTA 1 mM and betaine 0.5 M were used. ddPCR also allowed parallel DNA quantification. Amplifications were performed with the following thermal profile: 1 cycle at 95°C for 10 minutes, 40 cycles at 94°C for 30 seconds and 55°C for 1 minute (for C228T/C250T pTERT assays, 50 cycles at 96°C for 30 seconds and 62°C for 1 minute), followed by 98°C for 10 minutes. Positive-, negative- and no template controls were included in each run. For CNV analysis, the reaction included 1 × ddPCR supermix, primers and probes at a final concentration of 900 nM and 250 nM, respectively, and 2-15 ng of cfDNA for a total volume of 20 µl. The following thermal profile was applied: 1 cycle at 95°C for 10 minutes, 40 cycles at 94°C for 30 seconds and 60°C for 1 minute, followed by 98°C for 10 minutes. Data were analyzed by QuantaSoft analysis software version 1.7.4 (BioRad). BRAF and PTEN CNVs were assessed using as reference three different probes located on chromosomes 14, 7, and 10 (see Table A below).

Type of assay	Gene	Variant	Unique Assay ID	Sequence
**Multi target**	BRAF	V600E<br>V600K<br>V600R	dHsaMDV2010027<br>dHsaMDV2010035<br>dHsaMDV2010037	TCAGATATATTTCTTCATGAAGACCTCACAGTAAAAATAGGTGATTTTGGTCTAGCTACAG**[T/A]**GAAATCTCGATGGAGTGGGTCCCATCAGTTTGAACAGTTGTCTGGATCCATTTTGTGGATG<br>CTCAGATATATTTCTTCATGAAGACCTCACAGTAAAAATAGGTGATTTTGGTCTAGCTACA**[GT/AA]**GAAATCTCGATGGAGTGGGTCCCATCAGTTTGAACAGTTGTCTGGATCCATTTTGTGGATG<br>CTCAGATATATTTCTTCATGAAGACCTCACAGTAAAAATAGGTGATTTTGGTCTAGCTACA**[GT/AG]**GAAATCTCGATGGAGTGGGTCCCATCAGTTTGAACAGTTGTCTGGATCCATTTTGTGGATG
**Single target**	BRAF	V600E	dHsaCP2000027<br>dHsaCP2000028	TCAGATATATTTCTTCATGAAGACCTCACAGTAAAAATAGGTGATTTTGGTCTAGCTACAG**[T/A]**GAAATCTCGATGGAGTGGGTCCCATCAGTTTGAACAGTTGTCTGGATCCATTTTGTGGATG
**Single target**	BRAF	V600K	dHsaCP2000035<br>dHsaCP2000036	CTCAGATATATTTCTTCATGAAGACCTCACAGTAAAAATAGGTGATTTTGGTCTAGCTACA[**GT/AA]**GAAATCTCGATGGAGTGGGTCCCATCAGTTTGAACAGTTGTCTGGATCCATTTTGTGGATG
**Single target custom assay**	BRAF	T599dup	dHsaMDS440680521	CTCAGATATATTTCTTCATGAAGACCTCACAGTAAAAATAGGTGATTTTGGTCTAGCTACA**[-/ACA]**GTGAAATCTCGATGGAGTGGGTCCCATCAGTTTGAACAGTTGTCTGGATCCATTTTGTGGA
**Single target custom assay**	MEK1	P124L	dHsaMDS918405961	AAACCCGCAATCCGGAACCAGATCATAAGGGAGCTGCAGGTTCTGCATGAGTGCAACTCTC**[C/T]**GTACATCGTGGGCTTCTATGGTGCGTTCTACAGCGATGGCGAGATCAGTATCTGCATGGAG
**Single target**	NRAS	Q61L	dHsaMDS343494383	TACACAGAGGAAGCCTTCGCCTGTCCTCATGTATTGGTCTCTCATGGCACTGTACTCTTCT**[T/A]**GTCCAGCTGTATCCAGTATGTCCAACAAACAGGTTTCACCATCTATAACCACTTGTTTTCT
**Single target**	GNA11	Q209L	dHsaCP2000049<br>dHsaCP2000050	GGGGCGCCAGGTGGCTGAGTCCTGGCGCTGTGTCCTTTCAGGATGGTGGATGTGGGGGGCC**[A/T]**GCGGTCGGAGCGGAGGAAGTGGATCCACTGCTTTGAGAACGTGACATCCATCATGTTTCTC
**Single target custom assay**	TERT	C228T	dHsaEXD72405942	Corless *et al.*[2]
**Single target custom assay**	TERT	C250T	dHsaEXD46675715	Corless *et al.*[2]
**Single target**	FBXW7	R465C	dHsaMDV2510506	AGTGTGGAATGCAGAGACTGGAGAATGTATACACACCTTATATGGGCATACTTCCACTGTG**[C/T]**GTTGTATGCATCTTCATGAAAAAAGGTAAGGGAAAATCTTGTCATGGTTGGGACTTCTTCC
**Single target**	GNAQ	Q209L	dHsaCP2000051<br>dHsaCP2000052	TACTTTATATGTTATTAATATGAGTATTGTTAACCTTGCAGAATGGTCGATGTAGGGGGCC**[A/T]**AAGGTCAGAGAGAAGAAAATGGATACACTGCTTTGAAAATGTCACCTCTATCATGTTTCTA
**Single target**	TP53	S241F	dHsaMDV2516910	GTTATCTCCTAGGTTGGCTCTGACTGTACCACCATCCACTACAACTACATGTGTAACAGTT**[C/T]**CTGCATGGGCGGCATGAACCGGAGGCCCATCCTCACCATCATCACACTGGAAGACTCCAGG
**Single target**	TP53	R175H	dHsaMDV2010105	ACCCGCGTCCGCGCCATGGCCATCTACAAGCAGTCACAGCACATGACGGAGGTTGTGAGGC**[G/A]**CTGCCCCCACCATGAGCGCTGCTCAGATAGCGATGGTGAGCAGCTGGGGCTGGAGAGACGA
**Single target custom assay**	RAC1	P29S	dHsaMDS2513840	CTTTCTCTTTAGAGCTGTAGGTAAAACTTGCCTACTGATCAGTTACACAACCAATGCATTT**[C/T]**CTGGAGAATATATCCCTACTGTGTAAGTATCTTAAATTGGGAATTAACCTGTTTGTGTTAC
**Single target custom assay**	PIK3CA	E542K	dHsaMDS301083349	TGAATTAAGGGAAAATGACAAAGAACAGCTCAAAGCAATTTCTACACGAGATCCTCTCTCT**[G/A]**AAATCACTGAGCAGGAGAAAGATTTTCTATGGAGTCACAGGTAAGTGCTAAAATGGAGATT
**Single target**	PIK3CA	E545K	dHsaMDS986963652	GGAAAATGACAAAGAACAGCTCAAAGCAATTTCTACACGAGATCCTCTCTCTGAAATCACT**[G/A]**AGCAGGAGAAAGATTTTCTATGGAGTCACAGGTAAGTGCTAAAATGGAGATTCTCTGTTTC

**custom assay**				
**Single target custom assay**	PTEN	L247*	dHsaMDS152065706	TCCAATTCAGGACCCACACGACGGGAAGACAAGTTCATGTACTTTGAGTTCCCTCAGCCGT**[T/G]**ACCTGTGTGT GGTGATATCAAAGTAGAGTTCTTCCACAAACAGAACAAGATGCTAAAAAAG
**Single target custom assay**	RET	S705F	dHsaMDS214545579	GTCAGCTACTCCTCTTCCGGTGCCCGCCGGCCCTCGCTGGACTCCATGGAGAACCAGGTCT**[C/T]**CGTGGATGCC TTCAAGATCCTGGTGAGGGTCCCTGCGGGGCAGGGAAGATCCCCTGCCCTC
**Single target**	KRAS	G12A	dHsaMDV2510586	TTATTTTTATTATAAGGCCTGCTGAAAATGACTGAATATAAACTTGTGGTAGTTGGAGCTG**[G/C]**TGGCGTAGGC AAGAGTGCCTTGACGATACAGCTAATTCAGAATCATTTTGTGGACGAATAT
**Copy number assay**	BRAF	N/A	dHsaCP2500366	CCAATAGAGTCCGAGGCGGGTGCGGAAGGGGATGATCCAGATGTTAGGGCAGTCTCTGCTAAGGACGCCTCTT CCTGTGGTATTGGGTGGTGTTCAAAGAACTTGGAGACAAACAGCAAACTG
**Copy number assay (reference)**	TTC5	N/A	dHsaCP2506733	TGGTCGCGATGCCACTGTGGCAACAGCCTGGCTGCTGGATCCCTGAGGCTTCCCATTCACCACTAGCAGGAGGG GCGTCTCCACTCGAACACTGGAAAAGGAATAGTCCTAGAAAAGACAGAC
**Copy number assay (reference)**	VOPP1	N/A	dHsaCP2506684	TATGGAGAGGGCCCGCACACAGCACCTGGAGCCACAGCAGTCCTCGTAGGAGCGGCATCTGTGGAGAGAGGC ACAGGCTGGTCAGCACTGAATTGGAAGCAGCCACCGGACCAGCCATGCGGC
**Copy number assay (reference)**	RPP30	N/A	dHsaCP2500350	TCGGCCATCAGAAGGAGATGAAGATTGTCTTCCAGCTTCCAAGAAAGCCAAGTGTGAGGGCTGAAAAGAATGC CCCAGTCTCTGTCAGCACTCCCTTCTTCCCTTTTATAGTTCATCAGCCAC
**Copy number assay**	PTEN	N/A	dHsaCP2500323	CAAAAGGAGATATCAAGAGGATGGATTCGACTTAGACTTGACCTGTATCCATTTCTGCGGCTGCTCCTCTTTACC TTTCTGTCACTCTCTTAGAACGTGGGAGTAGACGGATGCGAAAATGTC

Table A. ddPCR assays and their specifications.

Custom NGS panel validation - Preparation of the sequencing library

The percentage of cfDNA was high for all samples, indicating a minimal presence of high molecular weight DNA. All samples displayed a typical shape of a cfDNA trace, with most specimens showing one nucleosome peak, and few having a smear in the region of the tri-nucleosome peak (Supplementary Figure 1). The library concentration was compared between Qubit and TapeStation. The varied amount of input cfDNA did not affect the final library concentration, as all libraries reported a quite similar concentration. A hybridization capture-based target enrichment custom panel (SureSelect Cancer All-In-One custom panel, Agilent Technologies) was used for the detection of SNVs and small deletions/insertions in 52 genes, plus CNV for 12 genes [1]. Its design covered hotspots for the driver and targetable mutations, together with the genes involved in the pathways associated with resistance to treatment and/or disease outcome for both cutaneous and uveal melanoma [3–10].

Libraries were prepared using the SureSelect XT HS Target Enrichment System (Agilent Technologies) according to the manufacturer’s instructions. Fragmentation was excluded due to the fragmented nature of cfDNA, and the number of PCR cycles was balanced to the median sample input amount (11 cycles). QC was performed after library preparation with the 4200 TapeStation System (High Sensitivity D1000 ScreenTape Assay, Agilent Technologies) to ensure quality libraries. The library concentration was compared between Qubit and TapeStation. The region table was set from 150 to 1000 bp and library sizes and concentrations were recorded. Library pools were prepared by combining an equal mass of each library and sequenced using the 300 cycle NextSeq 500/550 Mid Output v2 kit on the NextSeq 550 (Illumina).

CMC capture and enrichment

CMCs were enriched from 7.5 ml peripheral blood samples through the CellSearch system, using the Celltracks Circulating Melanoma Cell Kit (Menarini Silicon Biosystems, Bologna, Italy) that relies on CD146/HMW-MAA for capture and detection, according to the manufacturer’s instructions. A semi-automated fluorescence-based microscope system (CellTracks Analyzer II, Menarini Silicon Biosystems) was used to identify circulating melanoma cells. An event was classified as a CMC when its morphological features were consistent with those of a cell, and exhibited the phenotype CD146+, HMW-MAA+, DAPI+ and CD34/45- [11]. The DNA-damaged melanoma cells were identified by the integrated anti-γH2AX antibody (clone JBW301 from Merck KGaA, Darmstadt, Germany) that recognizes the phosphorylated form of histone H2AX (γH2AX), which is correlated to apoptotic chromatin fragmentation [12–15]. A CMC was classified as γH2AX-positive when the γH2AX signal co-localized with DAPI staining. Results are expressed as the total number of CMCs and γH2AX-positive CMCs per 7.5 mL of blood.

Collection, laser capture microdissection, WGA procedure and NGS

With the intent to investigate whether CMCs reflected spatial/temporal heterogeneity of disease, we developed a procedure for CMC recovery and subsequent DNA amplification. Cartridge content, recovered after the CellSearch run, was diluted in PBS and placed on the magnet for CMC retrieval. Resuspended cells were then deposited on a polyphenylene sulfide (PPS) membrane slide (Molecular Machines & Industries GmbH, Eching,

Germany), fixed and washed. Putative CMCs were finally detected under fluorescence guide, isolated by laser capture microdissection (MMI CellCut system, Molecular Machines & Industries GmbH - ECLIPSE Ti2 microscope, Nikon Corporation, Tokyo, Japan), and collected on a single-tube vial with adhesive cap (Molecular Machines & Industries GmbH). DNA from isolated cells was amplified using the Ampli1 WGA Kit (Menarini-Silicon Biosystems), subjected to QC by Ampli1 QC Kit (Menarini-Silicon Biosystems) and, if satisfactory, sent to Menarini-Silicon Biosystems for NGS analysis with the Ampli1 OncoSeek Panel, which is specifically designed to fit with the size of DNA fragments generated by the super amplification process.

Supplementary Material – References

1. Catoni, C. *et al.* Investigating the Retained Inhibitory Effect of Cobimetinib against p.P124L Mutated MEK1: A Combined Liquid Biopsy and in Silico Approach. *Cancers (Basel)* **14**, 4153 (2022).
2. Corless, B. C. *et al.* Development of Novel Mutation-Specific Droplet Digital PCR Assays Detecting TERT Promoter Mutations in Tumor and Plasma Samples. *J Mol Diagn* **21**, 274–285 (2019).
3. de Unamuno Bustos, B. *et al.* Towards Personalized Medicine in Melanoma: Implementation of a Clinical Next-Generation Sequencing Panel. *Sci Rep* **7**, 495 (2017).
4. Casula, M. *et al.* Germline and somatic mutations in patients with multiple primary melanomas: a next generation sequencing study. *BMC Cancer* **19**, 772 (2019).
5. Reiman, A. *et al.* Validation of an NGS mutation detection panel for melanoma. *BMC Cancer* **17**, 150 (2017).
6. Garman, B. *et al.* Genetic and Genomic Characterization of 462 Melanoma Patient-Derived Xenografts, Tumor Biopsies, and Cell Lines. *Cell Rep* **21**, 1936–1952 (2017).
7. Siroy, A. E. *et al.* Beyond BRAF(V600): clinical mutation panel testing by next-generation sequencing in advanced melanoma. *J Invest Dermatol* **135**, 508–515 (2015).
8. Afshar, A. R. *et al.* Next-Generation Sequencing of Uveal Melanoma for Detection of Genetic Alterations Predicting Metastasis. *Transl Vis Sci Technol* **8**, 18 (2019).
9. Versluis, M. *et al.* Digital PCR validates 8q dosage as prognostic tool in uveal melanoma. *PLoS One* **10**, e0116371 (2015).
10. de Lange, M. J. *et al.* Heterogeneity revealed by integrated genomic analysis uncovers a molecular switch in malignant uveal melanoma. *Oncotarget* **6**, 37824–37835 (2015).
11. Rao, C. *et al.* Circulating melanoma cells and survival in metastatic melanoma. *Int J Oncol* **38**, 755–760 (2011).
12. Rogakou, E. P., Nieves-Neira, W., Boon, C., Pommier, Y. & Bonner, W. M. Initiation of DNA Fragmentation during Apoptosis Induces Phosphorylation of H2AX Histone at Serine 139*. *Journal of Biological Chemistry* **275**, 9390–9395 (2000).
13. Bonner, W. M. *et al.* γH2AX and cancer. *Nat Rev Cancer* **8**, 957–967 (2008).
14. Garcia-Villa, A. *et al.* Assessment of γ-H2AX levels in circulating tumor cells from patients receiving chemotherapy. *Frontiers in Oncology* **2**, (2012).
15. Wang, L. H. *et al.* Monitoring Drug-Induced γH2AX as a Pharmacodynamic Biomarker in Individual Circulating Tumor Cells. *Clinical Cancer Research* **16**, 1073–1084 (2010).
